# A distributional multivariate approach for assessing performance of climate-hydrology models

**DOI:** 10.1038/s41598-017-12343-1

**Published:** 2017-09-21

**Authors:** Renata Vezzoli, Gianfausto Salvadori, Carlo De Michele

**Affiliations:** 1 Centro Euro-Mediterraneo sui Cambiamenti Climatici (CMCC), Regional Models and geo-Hydrological Impacts Division (REMHI), Capua (CE), I-81043 Italy; 2 Università del Salento, Dipartimento di Matematica e Fisica, Lecce, I-73100 Italy; 3 Politecnico di Milano, Dipartimento di Ingegneria Civile ed Ambientale (DICA), Milano, I-20133 Italy

## Abstract

One of the ultimate goals of climate studies is to provide projections of future scenarios: for this purpose, sophisticated models are conceived, involving lots of parameters calibrated via observed data. The outputs of such models are used to investigate the impacts on related phenomena such as floods, droughts, etc. To evaluate the performance of such models, statistics like moments/quantiles are used, and comparisons with historical data are carried out. However, this may not be enough: correct estimates of some moments/quantiles do not imply that the probability distributions of observed and simulated data match. In this work, a distributional multivariate approach is outlined, also accounting for the fact that climate variables are often dependent. Suitable statistical tests are described, providing a non-parametric assessment exploiting the Copula Theory. These procedures allow to understand (i) whether the models are able to reproduce the distributional features of the observations, and (ii) how the models perform (e.g., in terms of future climate projections and changes). The proposed methodological approach is appropriate also in contexts different from climate studies, to evaluate the performance of any model of interest: methods to check a model per se are sketched out, investigating whether its outcomes are (statistically) consistent.

## Introduction

Nowadays, world-wide climate simulations, performed under different IPCC emission scenarios, are available^1,2^ to the scientific community as inputs for impact/mitigation studies. Climate models represent a simplification of the climate system, and their outputs need to be validated against observations before their use in climate-change studies^3^, for example, to check the reliability of the calibration phase. Indeed, should this latter survey fail, then it would not be clear what the model projections would be representative of, and it would be difficult to interpret the (future) outputs of a model that is not even able to reproduce the relevant features of the observed data over which it has been calibrated^4,5^. In addition, it is also important to understand what has to be expected in terms of the features of the models’ outputs: for instance, whether or not, and how, the models are able to introduce changes of the (multivariate) probability distributions at play (viz., their ability to model possible climate changes), either at a marginal or at a joint level, or both.

Usually, specific statistics (of interest in the context of climate studies) are adopted to survey models’ performances. For example^6–14^, it is common to use overall indices, averages, variances, correlations, root-mean-square differences, monthly/annual averages, seasonal patterns, maximum and minimum values, quantiles, trends, etc. Now, the question is: *Is this proper and sufficient to claim that the models perform well?*

Rarely these analyses investigate^15^ the full distributional (probabilistic) features of the models, as well as the possible dependencies among the variables at play. In general, the fact that a model correctly reproduces some moments/quantiles of the observations (e.g., mean, variance, correlation, etc.), does not necessarily imply that also the whole (possibly multivariate) probability distribution of the data is matched. The compatibility of two distributions (viz., their statistical equality) is stronger than some moments/quantiles agreement: in fact, alikeness of some moments/quantiles is only a “weak” form of equality (it is a necessary condition for distributional equality, but not a sufficient one).

The case study presented here involves linked models: a climate model provides the inputs for a hydrological model, whose outputs are then used to extract time series of droughts in the region of interest, representing the hydrological response of the river basin considered. In order to provide a proper assessment of the performance of these models, suitable statistical tools, both univariate and multivariate, are outlined, and appropriate tests are carried out on the observations and the outputs of both the climate and the hydrological models, as well as on the drought data. Non-parametric diagnostic procedures, involving the probability distributions of the available samples, are introduced and discussed, both concerning the reliability of the calibration phase, and for understanding what are the probabilistic features of the (future) outputs of the models. In turn, two different (and complementary) upshots are presented and investigated: on the one hand, possible flaws of the models are “unveiled” via statistical techniques; on the other hand, possible failures of the statistical tests are considered and dealt with, and are “unmasked” via a logical construction of the sequence of analyses to be carried out.

It is important to stress that the choice of the specific models investigated in this work is of relative importance. In fact, here the target is not to judge whether the given models are good or bad. Rather, the paper is of methodological nature, and the goal is to provide statistical tools to evaluate the (distributional, i.e. probabilistic) features of *any* model of interest. Then, by integrating the results of traditional statistics/indices with those of the probabilistic analyses outlined in this work, the scientist may draw a thorough picture of the models’ performances, and decide whether (or not) to accept them as valuable descriptions of the phenomena under consideration. In particular, here the relationship between the climate model error and the hydrological model error (the linked models used in this work) are of no interest. In fact, the target is not to investigate possible “sources of errors”, or how errors combine/propagate from a model to another: this task can be carried out by the scientists in charge of the models if, and once, some of the statistical tests outlined in this work failed. Most importantly, as shown below, these tests give the possibility to (separately) examine whether, and where, errors may come up, either at the level of the climate model, or of the hydrological model, or both.

## Materials

The data considered in this study are extracted from observed and simulated climate-hydrology databases involving the Po river basin, the largest Italian one, covering an area of about 74,000 km^2^ from Western Alps to the Adriatic sea, with a main river length of about 650 km. The basin is an important economic area, producing about 40% of the national Gross Domestic Product: water uses involve strategic activities, such as agriculture, livestock, inland navigation, and industry. The observed and simulated data considered here are representative of the five main river sections of the Po river, the some ones used by the Control Room for water management: from East to West, Piacenza, Cremona, Boretto, Borgoforte, and Pontelagoscuro–see the corresponding Figure in the Supplementary Materials.

### Climate and Discharge Time series

The observed climate and discharge time series used in this work are taken from Hydrological Yearbooks (Part I and II): these are available online, or upon request from the Institute for Environmental Protection and Research^16^ (ISPRA) and Regional Agencies for Environment Protection^17–19^ (ARPAs), or from the Civil Protection Operative Center^20^ websites. In particular, time series of daily precipitation *P* (for the period 1971–2012, in *mm*), and 2 meter daily mean air temperature *T* (for the period 1990–2012, in °*C*), are obtained by interpolating (via the inverse of distance method) over a regular grid of 0.07° (the same one of the regional climate model used for simulations) the meteorological observations reported in Part I of Hydrological Yearbooks. Daily average discharges *Q* (for the period 1971–2012, in *m* ^3^ */s*) for the five gauge stations mentioned above are taken from Part II of Hydrological Yearbooks.

Time series of simulated *P*’s and *T*’s (viz., the outcomes^21^ of the climate model), covering the period 1982–2100, are considered. More specifically, the period 1982–2005 was simulated according to the CMIP5^1^ historical experiment, whereas the period 2006–2100 was simulated by forcing the climate model according to the IPCC RCP4.5 scenario^22^: this latter represents a stabilization scenario for which, in 2100, the global/overall radiative forcing will be about 4.5 W/m^2^ larger than in the pre-industrial era. Boundary conditions provided by CMIP5 and IPCC RCP4.5 are used to drive the global climate model CMCC-CM^23^, which, in turn, drives the regional climate model COSMO-CLM^24^ yielding the climatological time series. Then, precipitation (*P*) and air temperature (*T*) time series are extracted, and their systematic distortion^25^ is removed by applying a bias correction^10^ before performing hydrological simulations.

Simulated daily time series *Q*’s (viz., the outcomes^21^ of the linked climate-hydrology models), covering the period 1982–2100, are obtained by feeding the spatially distributed hydrological model TOPKAPI^26^ and the water balance model RIBASIM^27^ with the simulated *P*’s and *T*’s mentioned above. The time interval 1982–2005 is used as a control period for the simulated *P*'s, *T*'s, and *Q*’s.

**Figure 1:**
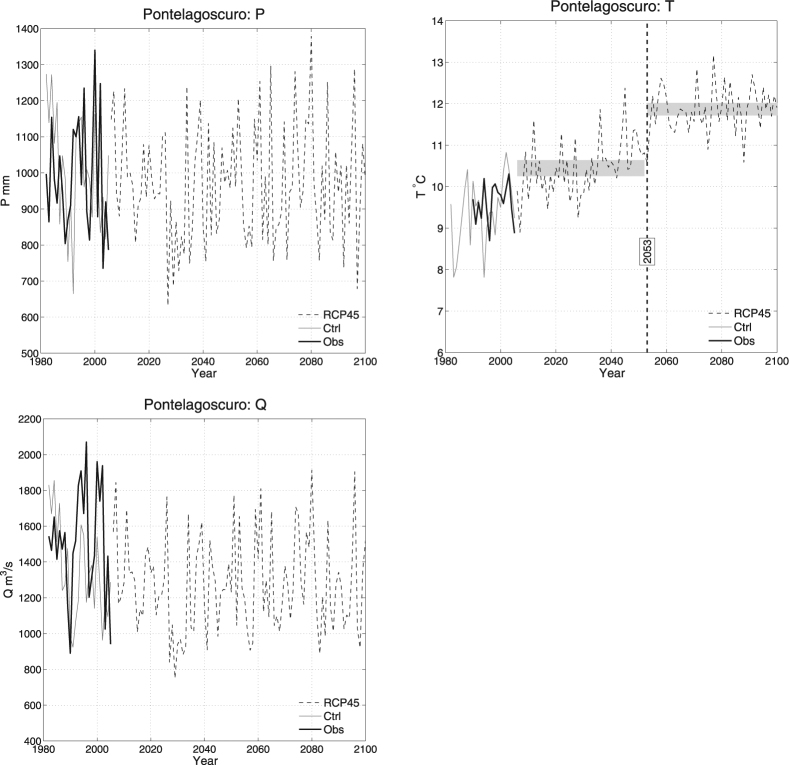
Time series of the variables *P* (*top-left* panel), *T* (*top-right* panel), and *Q* (*bottom-left* panel), corresponding to the following data sets at Pontelagoscuro: the Observations (*Obs*), the Control data (*Ctrl*), and the RCP4.5 data (*RCP45*). The *dashed* vertical line in the plot of the temperature indicates the possible change-point year (2053), and the *shaded* horizontal rectangles represent, respectively, empirical 95% confidence bands for the means of the data before and after the change-point.

As an illustration, Fig. 1 shows the available time series of the variables $P,T,Q$ at Pontelagoscuro corresponding to (i) the Observations over the period 1982–2005, (ii) the data simulated over the same time interval, and (iii) the data simulated according to the IPCC RCP4.5 scenario over the period 2006–2100. For the sake of brevity and conciseness, hereinafter only the case of Pontelagoscuro will be considered: in fact, this latter represents the most important river section for the Control Room, being the one closest to the outlet–the full set of results is reported in the Supplementary Materials.

### Drought time series

The hydrological response of the Po river basin to climate inputs is investigated through the analysis of the droughts measured at the five river sections mentioned above. Two main variables^28,29^ are used to characterize drought occurrences: namely, the duration *D* (in *days*), and the average intensity *I* (in *m* ^3^ */s*), viz. the average of all the daily differences between a suitable discharge threshold and the actual discharge. In particular, the so-called “${Q}_{300}$” threshold (considered as an “Alarm threshold” by the Po river Control Room), i.e. the discharge value that is exceeded at least 300 days per year–corresponding to the quantile of order ≈18% of the discharge distribution–is used here to identify the drought episodes of interest via a below-threshold analysis (also known as Run Method in hydrology)^30,31^: a minimum inter-event time of three days, and a minimum drought duration of five days, are used to (physically) circumvent^28^ possible dependencies between temporally successive droughts. According to the guidelines^32^ of the Control Room, such a procedure makes it possible to extract a statistically significant number of independent (extreme) droughts episodes–see the corresponding Table in the Supplementary Materials.

## Methods

In this section, statistical tools (mainly of distributional/probabilistic nature) for assessing the performances of the models of interest are outlined and discussed: the “R” software/routines used in this work are freely available on the Internet. Most importantly, the procedures outlined below are very general ones, and can be used to evaluate a broad family of models. For the ease of referencing, here the subsections are named with the same labels used later in the Results & Discussion section.

### Univariate analysis

A first fundamental step of the investigation procedure consists in considering the univariate distribution functions of the variables at play. The statistical tests outlined below may provide important information about the features of such probability laws.

- **Homogeneity test**. A first issue of interest concerns the test whether the univariate distributions of the observations are matched by the ones of the simulated variables, both over the control period and the future temporal horizon. Among others, basic procedures to carry out such a task are the non-parametric Kolmogorov-Smirnov^33^ (KS) and Anderson-Darling^34^ (AD) homogeneity tests. Here, the homogeneity assumption is “${ {\mathcal H} }_{0}$: the (univariate) samples come from the same distribution”. Note that the KS test is more powerful concerning the body of the (unknown) distribution, while the AD one is more specific for the tails: the two tests are different and complementary, and both should be used.
- **Change-Point test**. A further important issue concerns the test whether the univariate distributions of interest show Change-Points, i.e. whether they may (statistically) change with time: this latter case might be interpreted as a fingerprint of a possible climate change. Given the generic variable *X*, with distribution *F*_*X*_, the Change-Point assumption is “${ {\mathcal H} }_{0}$: *F*_*X*_ does not change with time”: roughly, a stationarity statement.

### Multivariate analysis: Copulas and joint distributions

A simple and elegant way to carry out a proper multivariate investigation concerning the joint probability distribution of the variables at play consists in exploiting the representation result provided by Sklar’s Theorem^35^. Briefly, it states that any multivariate law ${{\bf{F}}}_{{X}_{1},\ldots ,{X}_{d}}$ can be written as, for all $({x}_{1},\,\ldots ,\,\,{x}_{d})\in {{\bf{R}}}^{d}$,

1 $${{\bf{F}}}_{{X}_{1},\ldots ,{X}_{d}}({x}_{1},\ldots ,{x}_{d})={\bf{P}}({X}_{1}\le {x}_{1},\ldots ,{X}_{d}\le {x}_{d})={{\bf{C}}}_{{X}_{1},\ldots ,{X}_{d}}({F}_{1}({x}_{1}),\ldots ,{F}_{d}({x}_{d})),$$

where the ${F}_{i}$'s are the univariate marginals of the random variables ${X}_{i}$’s of interest, and ${{\bf{C}}}_{{X}_{1},\ldots ,{X}_{d}}$ is their copula^36–39^. In simple words, the copula function provides the mathematical structure modeling (only) the links between the variables. For instance, suppose that the random variables *Y* and *Z* are *concordant* (and, hence, dependent): viz., small/large values of *Y* are likely to be associated with small/large values of *Z*, and vice-versa. Then, e.g. during a bivariate simulation of (*Y*,*Z*), the corresponding copula acts on the functional expression of the joint distribution **F**_*YZ*_ in order to “promote/associate” the occurrence of small/large values of *Y* with small/large values of *Z*: as an example, the Gumbel-Hougaard copula^36,37^, frequently used in applications, operates in such a way. Note that copulas may also model two extreme instances^36,37^: (*i*) the case of independent variables (actually, independence is just a “special case” of dependence), and (*ii*) the case of variables functionally (i.e., deterministically) related. Most importantly, any modification introduced in the expression of **C** only affects **F** and not the ${F}_{i}$'s (since these latter are simply the arguments of **C** in Eq. (1)); similarly, changes in the marginals ${F}_{i}$'s modify **F** but leave **C** unaltered. As will be shown below, these features are of fundamental importance for the assessment of a model’s performance.

In turn, several interesting (multivariate) statistical tests can be performed: in particular, the following distributional/probabilistic issues are of utmost importance in climate studies and model’s validation, and the analysis via Copulas gives the possibility to spot where similarities and/or differences are present, and draw sensible conclusions.

- **Homogeneity test**. Check whether the (multivariate) homogeneity assumption “${ {\mathcal H} }_{0}$: ${{\bf{C}}}_{{\rm{data}}}$ and ${{\bf{C}}}_{{\rm{model}}}$ are the same” should be rejected or not. Here, ${{\bf{C}}}_{{\rm{data}}}$ and ${{\bf{C}}}_{{\rm{model}}}$ represent, respectively, the copulas (i.e., the dependence structures) associated with the observations and the data generated by the climate-hydrology model considered. Non-parametric procedures^40,41^ for testing the equality between copulas have recently been made available. Thanks to Sklar’s Theorem, it is then possible to provide a (statistical) answer to the more general question “${{\bf{F}}}_{{\rm{data}}}$ and ${{\bf{F}}}_{{\rm{model}}}$ are the same?”, of interest in climate investigations: in fact, it is enough to check whether both the corresponding marginals (via the univariate homogeneity tests mentioned above) and copula are (statistically) the same, otherwise the homogeneity assumption ${{\bf{F}}}_{{\rm{data}}}\equiv {{\bf{F}}}_{{\rm{model}}}$ should be rejected.
- **Change-Point test**. Check whether the (multivariate) Change-Point assumption “${ {\mathcal H} }_{0}$: **F** has no change-points” (viz., **F** does not change with time) should be rejected or not. A statistical answer to the fundamental question “Is a climate change present?” could then be given via Sklar’s representation by checking^42,43^ whether ${ {\mathcal H} }_{0}$ should be rejected or not, by investigating whether either the marginals, or the copula, or both, show change-points.

### Multivariate analysis: association measures

Quite often, climate variables are not independent, and the Pearson linear correlation coefficient ${\rho }_{P}$ is traditionally used to quantify their degree of association. However, ${\rho }_{P}$ only measures the linear relation between two variables, it depends upon the marginal distributions, and may not exist when their second order moments are not finite. Instead, the (non-parametric) rank-based approaches suggested here, exploiting the Kendall *τ*_*K*_ and the Spearman *ρ*_*S*_ statistics^36,37^, do not suffer from these problems: in fact, these measures of association always exist, and do not depend upon the marginals, but only on the copula at play (and, thus, provide an insight into the dependence structure of interest). In particular^36,37^, *τ*_*K*_ measures the excess of concordance/discordance in the sample, while *ρ*_*S*_ is proportional to the average difference between the copula of the data and the one modeling independent variables. In turn, *τ*_*K*_ provides information about the “likelihood” of small/large values of a variable *Y* given that the other variable *Z* is small/large (and vice-versa), while *ρ*_*S*_ measures how “far” is the joint behavior of $(Y,Z)$ from the one of independent pairs. Clearly, a valuable multivariate model should be able to reproduce the values of *τ*_*K*_ and *ρ*_*S*_ estimated for the data considered. Furthermore, both *τ*_*K*_ and *ρ*_*S*_ can be used to carry out non-parametric independence tests: if their values are not significantly different from zero (at some chosen level), then the independence assumption cannot be rejected. Such tests should always be carried out, in order to understand whether a multivariate approach makes sense and is worth. Note that, in general, the estimates of *τ*_*K*_ and *ρ*_*S*_ are robust against small sample sizes.

### Multivariate analysis: dependence structures

In case the independence among the variables at play is (statistically) rejected, it is then important to check whether the model of interest is able to reproduce the dependence structures (i.e., the copulas) of the data. A preliminary visual survey of the dependence structure linking the variables can be carried out by analysing the scatter-plots of the pseudo-observations^44^, viz. the plots of the data ranks normalized into the interval (0, 1), a non-parametric tool frequently used in multivariate analysis to visualize the main features of the dependence structure of interest. By construction, the pseudo-observations are not affected by the behavior of the marginals (the ranks are independent of the distribution of a variable): actually, they only depend upon the copula at play, and may provide specific information about the dependence structure ruling the joint dynamics. Although only of visual interest, these graphs represent a simple way to get a hint of the copula involved: for instance, they may indicate whether positive/negative associations, or lower/upper tail dependencies^37^, are present. Beyond the visual approach, objective statistical analyses can be carried out via, e.g., the homogeneity tests mentioned above. Clearly, if a model is unable to reproduce the distributions of the observations of interest (viz., the ones over which it has been calibrated), then the model outputs and the corresponding (future) byproducts may not be reliable and/or difficult to interpret, since the (probabilistic) structures and links imposed by the model on the variables at play may be different and/or unrelated to the observed ones.

### Treatment of repeated observations

A final important issue concerns the particular structure of some data bases, which may adversely affect both the univariate and the multivariate analyses. Sometimes, the procedures used to collect the data may introduce repeated values (called Ties), essentially due to lack of instrumental resolution and/or sampling strategies: for instance, this may be the case of droughts^28^ and sea storms^45^. In turn, continuous variables are transformed into discrete ones, which may have a non-negligible impact on the rank-based inference of copulas and univariate/multivariate probability distributions^46–49^, and may spoil the results of the statistical investigations, which may become difficult to interpret.

A possible way to circumvent the problem is to use appropriate randomization techniques^49^: this may turn the (discrete) repeated values into (continuous) different ones. The idea is to add to all the repeated values a set of suitable random perturbations in the range of the instrumental resolution adopted during the data sampling: in order to avoid unverifiable assumptions, the noise is chosen to be Uniform (i.e., a least-informative approach). Apparently, according to the results shown in refs ^28,45^, the randomization procedures may not significantly affect the original statistical structure of the data, while reducing the nuisance possibly introduced by Ties. In this work, ${N}_{R}=200$ independent randomizations are carried out for drought data, the only ones showing Ties.

## Results and Discussion

In this section, the results of several univariate and multivariate analyses are presented and discussed, involving both climate and hydrological variables. Here, three data sets are considered:

1. the Observations (labeled “Obs”), viz. the actual data collected from 1982 to 2005 (the “control” period);
2. the Control data (labeled “Ctrl”), viz. the data simulated from 1982 to 2005;
3. the future projections (labeled “RCP45”), viz. the data simulated from 2006 to 2100 under the IPCC RCP4.5 scenario.

The outcomes shown later may answer (at least) three important questions.

- *Is the model able to reproduce the probability distributions of the observed data over which it has been calibrated?* (alternatively, put in other words, Are the probability distributions of the data simulated over the control period different from the ones of the observed data?) Should the check fail, then a flaw in the model would be likely, and it would not be clear what the model outputs (both during the control period and the future temporal horizon) would be representative of. Evidently, a positive answer to this question represents a necessary condition for the internal coherence and consistency of the models at play.
- Assuming that the answer to the previous question is positive, viz. the probability distributions of observed and simulated data over the control period are (statistically) compatible, the following query is a natural one: *Are the probability distributions of the data simulated over the control period different from the ones of the future projections?* Should the answer be positive, then forcing the model according to the IPCC RCP4.5 scenario (or any other “recasting” mechanism) might effectively change the distributional/probabilistic features of the simulated variables.
- Should the future (simulated) projections feature a probability distribution (statistically) different from the one of the data simulated over the control period, a further important enquiry is as follows: *Are the probability distributions of the future projections different from the ones of the past observation?* A negative answer to this question might reveal possible flaws of the statistical techniques adopted. In fact, if the observations and the simulations over the control period were compatible (as it should be for any reliable/consistent model calibrated on the observed data), whereas the data simulated over the control period were incompatible with the future projections, then only a failure of the statistical tests might explain the possible (distributional/probabilistic) compatibility between the observed data and the ones simulated over a future temporal horizon. Practically, from a logical point of view, the observations cannot have, at the same time, the incompatible distributions of the control and the future simulated data, unless some of the tests failed. In other words, the distribution of the data simulated over the control period and the distribution of the future projections cannot be, at the same time, statistically incompatible with one another, but both statistically compatible with the distribution of the observations.

### Univariate analysis: homogeneity tests

**Figure 2:**
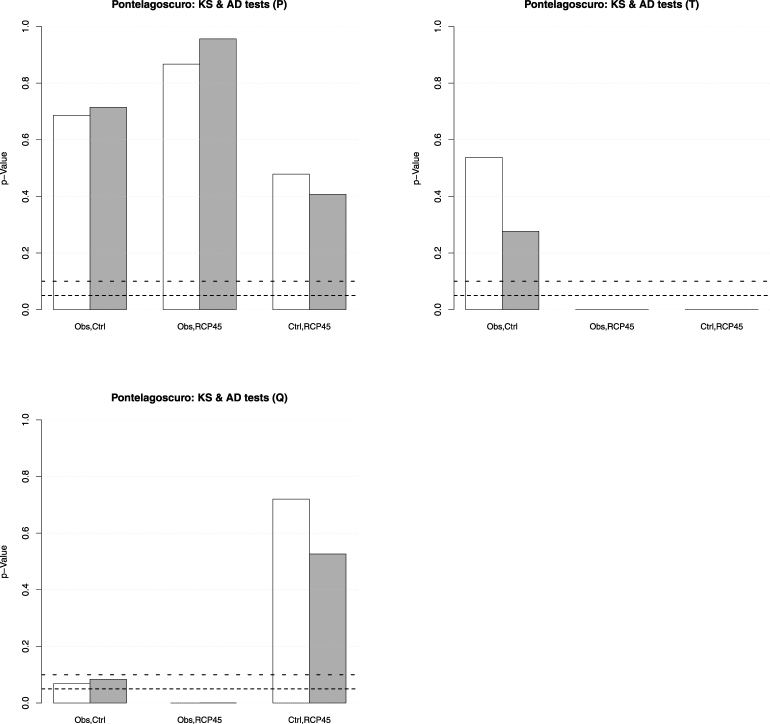
*p*-Values of the (univariate) two-sample Kolmogorov-Smirnov (*white* bars) and Anderson-Darling (*gray* bars) homogeneity tests, for the variables *P* (*top-left* panel), *T* (*top-right* panel), and *Q* (*bottom-left* panel), corresponding to the following pairs of data sets at Pontelagoscuro: the Observations–Control (*Obs,Ctrl*), the Observations–RCP4.5 (*Obs,RCP45*), and the Control–RCP4.5 (*Ctrl,RCP45*). The test indicates whether the Null hypothesis “${ {\mathcal H} }_{0}$: the (univariate) samples come from the same distribution” should be rejected at some given level: the *dashed* horizontal lines correspond to the 5% (*fine*) and the 10% (*coarse*) reference levels, respectively.

A first study of interest concerns the comparison between the univariate distributions of the variables $P,T,Q$ considering (*i*) the observed data, (*ii*) the data simulated over the control period, and (*iii*) the future projections. Figure 2 shows the *p*-Values of the Kolmogorov-Smirnov (KS) and Anderson-Darling (AD) homogeneity tests at Pontelagoscuro. Considering yearly mean values, the following facts are evident.

- **The variable** *P*. All the distributions are statistically compatible, both considering the body and the tails of the (unknown) probability laws at play. Thus, the climate model correctly reproduces the behavior of the observed yearly Precipitation over the control period, and replicates it unaltered over the future temporal horizon (i.e., no IPCC RCP4.5 effects are introduced/detected, which may (or may not) be desirable).
- **The variable** ***T***. Only the distributions of the observed and control data are statistically compatible, considering both the body and the tails of the (unknown) probability laws at play. Instead, the homogeneity assumption has to be rejected considering the future projections. In turn, apparently, forcing the climate model according to the IPCC RCP4.5 scenario might induce some changes concerning the probability distribution of future temperatures–see also later.
- **The variable** ***Q***. Here, the matter is unclear. In fact, considering the observed data and the future projections, the homogeneity assumption should definitely be rejected, whereas the distributions of the data simulated over the control period and the future projections turn out to be statistically compatible. In turn, one would expect that also the observed and the control data were not compatible, but in this case the statistical test indicates that the homogeneity assumption (both for the body and the tails of the (unknown) probability laws at play) should not be rejected at a 5% level, whereas the test fails at the 10% one. Thus, possible interpretations are as follows.

1. The statistical indication of accepting the compatibility between the probability distributions of observed and control data at a 5% level is wrong (i.e., a failure of the test due, e.g., to lack of power), and actually such a homogeneity assumption should be rejected. In turn, the climate-hydrology models would be unable to correctly reproduce the distributional behavior of the observed Discharge over the control period, and such “inability” is simply replicated over the future temporal horizon, which may represent a flaw of the models.
2. The supposed compatibility between control data and future projections is wrong. In turn, it is the statistical test that fails to suggest the rejection of the statistical/probabilistic compatibility between control data and future projections. Thus, forcing the models according to the IPCC RCP4.5 scenario might effectively introduce changes of the Discharge distribution.

Which of the two interpretations is the correct one (if any) is difficult to say.

**Figure 3:**
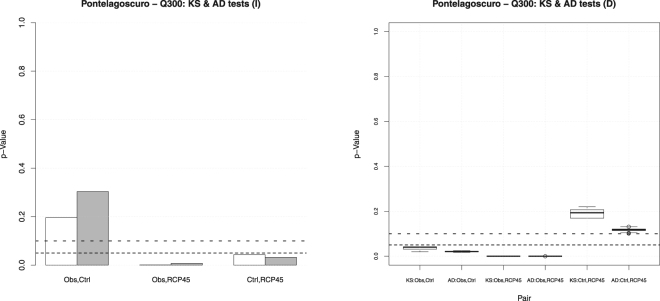
*p*-Values of the (univariate) two-sample Kolmogorov-Smirnov (*white* bars) and Anderson-Darling (*gray* bars) homogeneity tests, for the variables *I* (*left* panel), and *D* (*right* panel), and the threshold ${Q}_{300}$ at Pontelagoscuro, corresponding to the following pairs of data sets: the Observations–Control (*Obs,Ctrl*), the Observations–RCP4.5 (*Obs,RCP45*), and the Control–RCP4.5 (*Ctrl,RCP45*). The *dashed* horizontal lines correspond to the 5% (*fine*) and the 10% (*coarse*) reference levels, respectively. Since Ties are present for *D*, the corresponding box-plots are over all the *N*_*R*_ randomizations–see text.

Considering the time series of drought episodes, based on daily discharge values, the results are shown in Fig. 3.

- **The variable** *I*. The distributions of observed and control data are statistically compatible, considering both the body and the tails of the (unknown) probability laws at play. Instead, the homogeneity assumption has to be rejected (at a 5% level) considering the future projections. In turn, apparently, forcing the linked climate-hydrology models according to the IPCC RCP4.5 scenario might change the probability distribution of the drought intensities.
- **The variable** *D*. Apparently, the distribution of the observed data are statistically incompatible (considering both the body and the tails of the (unknown) probability laws at play, at a 5% level) with those of the control data and the future projections. Instead, the homogeneity test is passed by these two latter distributions. The results are unvarying over all the ${N}_{R}$ randomizations, as indicated by the corresponding box-plots. Thus, apparently, according to the model projections, the future drought durations are expected to show a probability distribution different from the one of the observed data. However, as for the case of the variable *Q* discussed above, it is not clear whether this is due to the fact the linked climate-hydrology models might be unable to reproduce the behavior of *D* over the control period, and such “inability” is simply replicated over the future temporal horizon: this may represent a flaw of the models.

Provided that the available sample sizes are large enough, a further important information that could be drawn via the KS and AD tests is as follows. As a matter of principle, it might happen that the homogeneity assumption should be rejected according to the KS test (involving the body of the distribution), but it should not be according to the AD one (involving the tails of the distribution), and/or vice-versa. As an example, see the case of the gauge station Borgoforte for the variable *D* reported in the Supplementary Materials, considering the comparison between the control and the future distributions: here the sample sizes are larger than 60, and hence sensible statistical conclusions might be drawn. Apparently, the distribution of the data simulated over the control period is statistically compatible (at a 5% level) with the one of the future projections considering the body of the probability law (as a result of the KS test), but it may be incompatible considering the tails of the distribution (as a result of the AD test). Such a piece of information may be quite important: on the one hand, it reveals how the model behaves, and what kind of changes it may introduce; on the other hand, should it be desirable, it provides useful indications to precisely adjust the model.

For the sake of clarity, the results concerning the univariate Change-Point tests are discussed later, in the corresponding Section dealing with the multivariate analysis: in fact, due to the Sklar’s representation of the joint distribution ${{\bf{F}}}_{{X}_{1},\ldots ,{X}_{d}}$ provided by Eq. (1), it is better to examine together the behaviors of the marginals and the copula.

### Multivariate analysis: association measures

**Figure 4:**
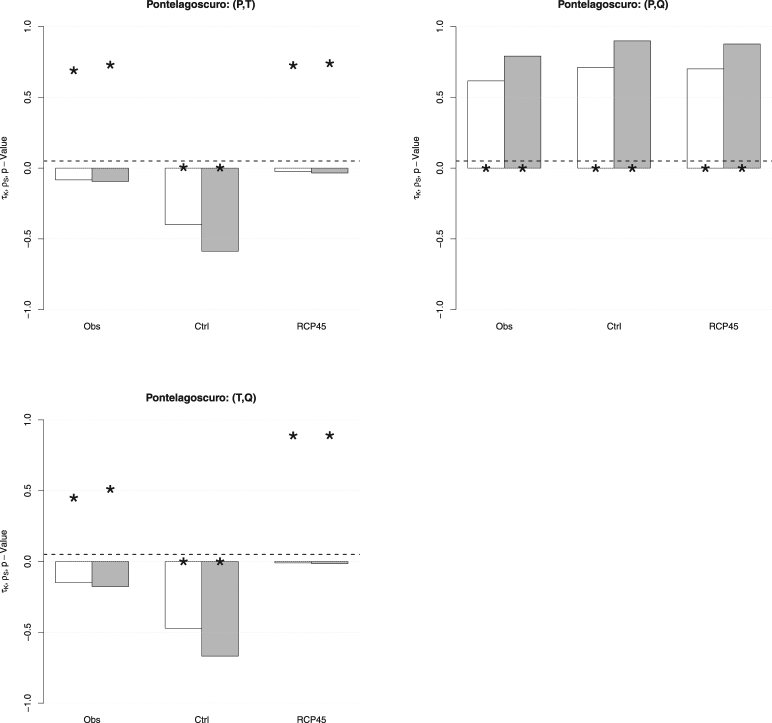
Estimates of the Kendall *τ* (*white* bars) and the Spearman $\rho$ (*gray* bars), as well as the corresponding *p*-Values (*star* markers), for the pairs $(P,T)$ (*top-left* panel), $(P,Q)$ (*top-right* panel), and $(T,Q)$ (*bottom-left* panel), corresponding to the following data sets at Pontelagoscuro: Observations (*Obs*), Control (*Ctrl*), and RCP4.5 (*RCP45*). The *dashed* horizontal line corresponds to the 5% reference level.

As explained in the Methods Section, a simple (non-parametric) way to understand whether a multivariate approach makes sense consists in calculating suitable rank-based association measures such as the Kendall *τ*_*K*_ and the Spearman *ρ*_*S*_ statistics, as well as the corresponding *p*-Values. Considering the variables $P,T,Q$, the results are shown in Fig. 4.

- **The pairs** (*P*, *T*) and (*T*, *Q*). In both cases, only the pairs simulated over the control period are significantly negatively associated, being the *p*-Values negligible. In turn, apparently, the model introduces a dependence between *P* and *T*, and *T* and *Q*, in the control period that is neither present in the original observations nor in the future projections. Such a behavior is difficult to interpret and explain: seemingly, it may represent a flaw of the model, and no sensible indications about what is likely to be expected in the future can be achieved.
- **The pair** (*P*, *Q*). A significant positive association, with about the same magnitude, is present in all cases. Here the model correctly reproduces the concordance between Precipitation and Discharge during the control period, and replicates it in the future projections. Accordingly, no changes in the degree of association between *P* and *Q* have to be expected over the future temporal horizon (i.e., no IPCC RCP4.5 effects are introduced, which may (or may not) be desirable).

It is worth stressing that, even if in some cases the estimates of *τ*_*K*_ and/or *ρ*_*S*_ might look, at first glance, numerically “fairly” different from zero (and, hence, the corresponding pair of variables should to all appearances be considered as dependent), only the *p*-Value may objectively quantify to what extent the independence assumption should be rejected. For instance, this is the case of the pairs $(P,T)$ and $(T,Q)$, for which, respectively, ${\tau }_{K},{\rho }_{S}\approx -0.1$ and ${\tau }_{K},{\rho }_{S}\approx -0.2$ (rather different from zero): however, the corresponding *p*-Values are (much) larger than 5%, and thus there is no (statistical) reason to reject the hypothesis of independence between *T* and the other two variables.

**Figure 5:**
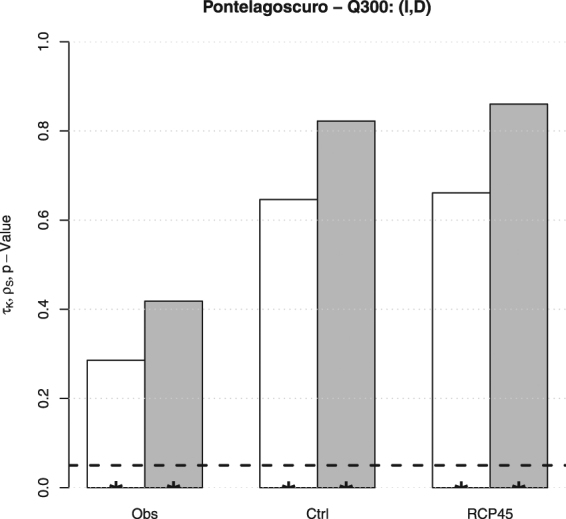
Estimates of the Kendall *τ* (*white* bars) and the Spearman *ρ* (*gray* bars), as well as the corresponding *p*-Values (*star* markers), for the pair $(I,D)$ and the threshold ${Q}_{300}$ at Pontelagoscuro, corresponding to the following data sets: Observations (*Obs*), Control (*Ctrl*), and RCP4.5 (*RCP45*). The *dashed* horizontal line corresponds to the 5% reference level.

Considering droughts, the results are shown in Fig. 5. Here, *I* and *D* are significantly positively associated in all cases. However, the simulated data show a degree of concordance that is about two times the one estimated for the observed data, a non-negligible increase. The fact that the actual degree of dependence between *I* and *D* is not reproduced during the control period may represent a flaw of the model, which is possibly incorrectly replicated over the future temporal horizon.

### Multivariate analysis: dependence structures

**Figure 6:**
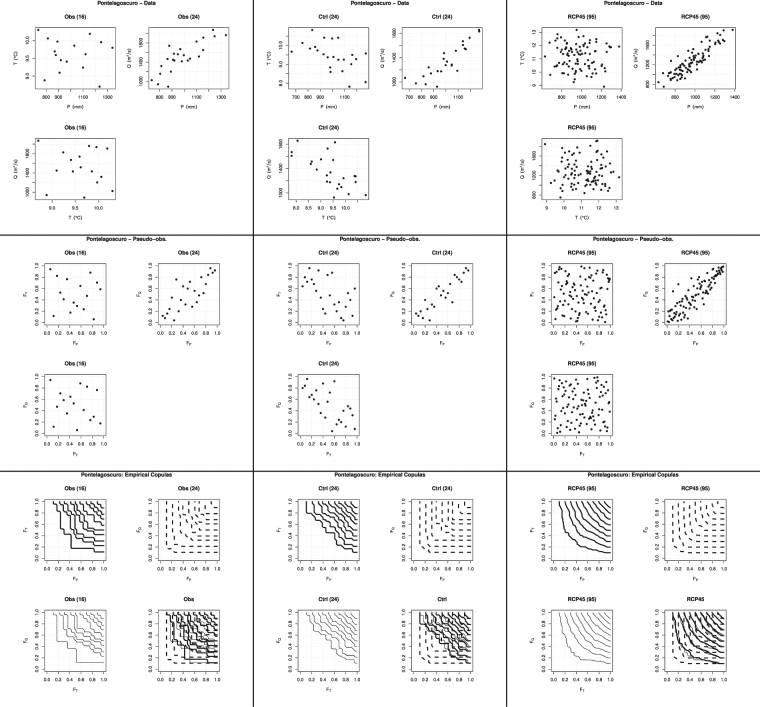
Here, the *left* panels concern the Observations (*Obs*), the *middle* panels concern the Control data (*Ctrl*), and the *right* panels concern the RCP4.5 data (*RCP45*): also reported are the sample sizes (in parentheses). (*Top* panels) Plotted are the data of the pairs $(P,T)$, $(P,Q)$, and $(T,Q)$. (*Middle* panels) Plotted are the pseudo-observations of the pairs $(P,T)$, $(P,Q)$, and $(T,Q)$. (*Bottom* panels) Plotted are the isolines of the Empirical Copulas of the pairs $(P,T)$, $(P,Q)$, and $(T,Q)$, as well as an overall comparison: the levels are $\mathrm{0.1:0.1:0.9}$.

A visual inspection of the available measurements is traditionally carried out via the scatter-plots of the data: these are shown in Fig. 6(*Top* panels) for the variables $P,T,Q$. According to Sklar’s Theorem (see Eq. (1)), these pictures are the by-product of the link induced by a copula ${{\bf{C}}}_{{X}_{1},\ldots ,{X}_{d}}$ over some marginals ${F}_{i}$'s, resulting in a joint distribution ${{\bf{F}}}_{{X}_{1},\ldots ,{X}_{d}}$. While graphical estimates of the univariate marginals can be calculated via (non-parametric) empirical distribution functions (not shown), the structure of the underlying copula can be guessed by plotting the corresponding (non-parametric) pseudo-observations, as anticipated in the Methods Section and shown in Fig. 6(*Middle* panels). As previously pointed out, the pseudo-observations are not affected by the marginals (they only depend upon the copula), and may provide a hint of the dependence structure at play.

The results of the Kendall and Spearman tests mentioned above find a graphical visualization in Fig. 6(*Middle* panels). In fact, the pseudo-observations of the pairs $(P,T)$ and $(T,Q)$ are essentially uniformly distributed in the unit square (or slightly aligned along the secondary diagonal), in agreement with the previously claimed independence (or apparent negative association) between the variables. Instead, in the $(P,Q)$ case, the markers well lie along the main diagonal, indicating the presence of a positive association (viz., concordance) between *P* and *Q*.

The *Bottom* panels of Fig. 6 show the isolines of the Empirical Copulas of interest (viz., the non-parametric estimates^44^ of the true, but unknown, dependence structures), which provide a further graphical support to the results of the statistical tests mentioned above. In fact, while the isolines of the pairs $(P,T)$ and $(T,Q)$ closely resemble those of the copula modeling independent variables, the ones of the $(P,Q)$ pair are similar to those of the copula modeling a strong association/concordance between the variables of interest (for a graphical comparison, see e.g. Figure 2.2 in ref. ^36^).

**Figure 7:**
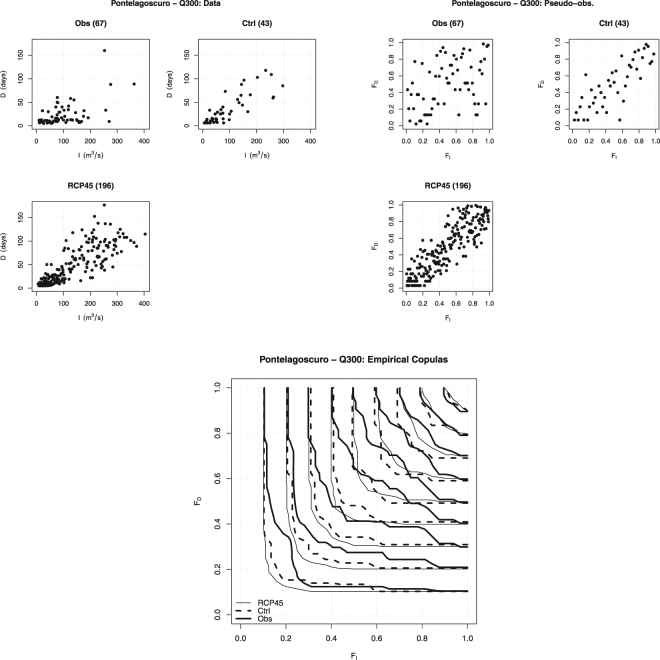
Observed $(I,D)$ pairs (*Top-left* panel) and Pseudo-observations (*Top-right* panel), for the threshold ${Q}_{300}$ at Pontelagoscuro, corresponding to the following data sets: Observations (*Obs*), Control (*Ctrl*), and RCP4.5 (*RCP45*). Also reported are the sample sizes (in parentheses). The *Bottom* panel shows the isolines of the Empirical Copulas, averaged over all the ${N}_{R}$ randomizations, for the levels $\mathrm{0.1:0.1:0.9}$.

Similar conclusions can be drawn considering the plots in Fig. 7, concerning the pair $(I,D)$. In particular, the fact that the pseudo-observations are (to different extents) aligned along the main diagonal is in agreement with the previously claimed different degree of concordance (viz., positive association) between drought Intensity and Duration. The shapes of the isolines of the Empirical Copula of interest are in agreement with the conclusions previously drawn.

### Change-Point and Copula-equality tests

The ability of the linked climate-hydrology models to introduce changes in the probability distributions of the relevant variables (over a temporal domain of interest), both at a univariate and at a multivariate level, can be investigated via suitable (non-parametric) statistical tests: clearly, these studies may provide important information about the features and the behavior of the models (like, e.g., the possibility to account for possible climate changes).

**Figure 8:**
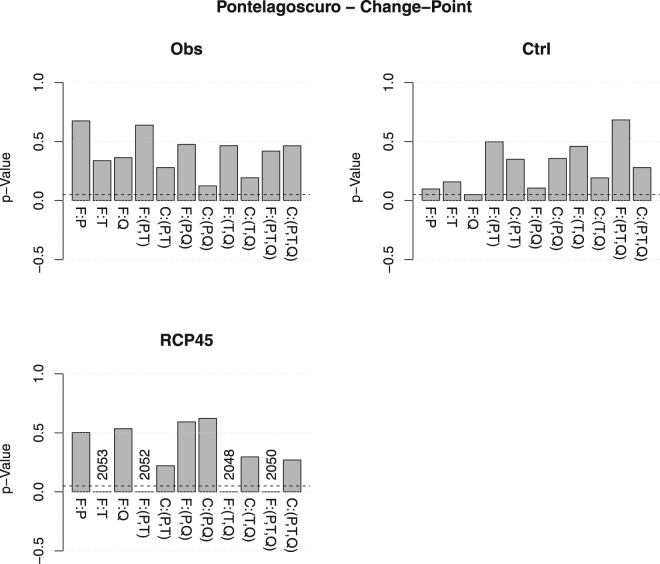
*p*-Values of the Change-Point test, corresponding to the following data sets at Pontelagoscuro: Observations (*Obs*), Control (*Ctrl*), and RCP4.5 (*RCP45*). The distributions considered are (from left to right): the marginal of the Precipitation *P* (*F:P*), the marginal of the Temperature *T* (*F:T*), the marginal of the Discharge *Q* (*F:Q*), the joint distribution of $(P,T)$ (*F:*(*P,T*)), the copula of $(P,T)$ (*C:*(*P,T*)), the joint distribution of $(P,Q)$ (*F:*(*P, Q*)), the copula of $(P,Q)$ (*C:*(*P*, *Q*)), the joint distribution of $(T,Q)$ (*F:*(*T*, *Q*)), the copula of $(T,Q)$ (*C:*(*T*, *Q*)), the joint distribution of $(P,T,Q)$ (*F:*(*P*, *T*, *Q*)), and the copula of $(P,T,Q)$ (*C:*(*P*, *T*, *Q*)). The *dashed* horizontal line corresponds to the 5% reference level. The *vertical* labels indicate possible change-point years.

Figure 8 shows the results of Change-Point tests for the variables $P,T,Q$, and all the distributions of interest (univariate, multivariate, and copulas). Apparently, at a 5% level, no change-points are present in the observed data, as well as in the data simulated during the control period. Instead, to all appearances, forcing the model according to the IPCC RCP4.5 scenario might introduce some changes in the distribution *F*_*T*_ of the temperature *T* (see also above) around the period 2048–2053. In turn, according to the Sklar’s Theorem representation of a multivariate distribution (see Eq. (1)), it is enough that either the marginals, or the copula, change in order to affect ${{\bf{F}}}_{PT}$, ${{\bf{F}}}_{TQ}$, and ${{\bf{F}}}_{PTQ}$ (viz., all the probability laws involving *T* may undergo some change), as is clearly shown in the bar-plot of the *p*-Values. As a matter of fact, considering the temperature data shown in Fig. 1(*top-right* panel), apparently the means of the samples simulated under the IPCC RCP4.5 scenario before and after 2053 (the possible change-point year for the distribution of *T*) are statistically different: the *p*-Value of the corresponding mean-equality test is negligible. Actually, it is also visually clear that the 95% confidence bands for the means of the data before and after the change-point are definitely distinct and non-overlapping, and the plot indicates a future increase of the mean. However, such an analysis would not have been possible without the preliminary indication of the break-point year (2053) provided by the Change-Point test, which may help in spotting and identifying possible climate changes.

A final important point is that, in all cases, the copulas at play show no change-points: in practice, apparently the models considered may only affect the “temporal” structure of the marginals, leaving the dependence structures unaltered. This represents a feature of the models, which may (or may not) be desirable when the IPCC RCP4.5 forcing is introduced.

**Figure 9:**
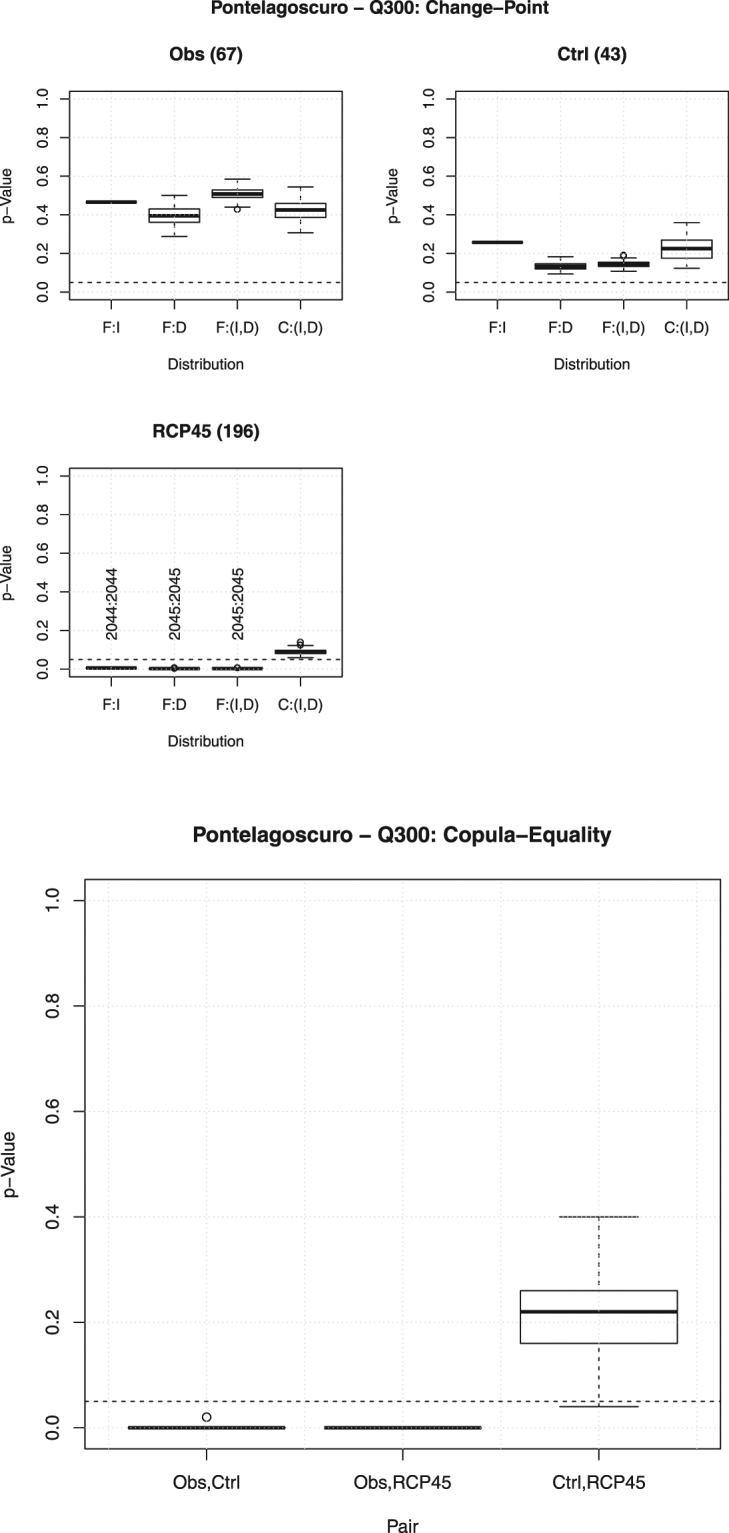
Box-plots of the *p*-Values of the Change-Point test (*Top* panel) and of the Copula-Equality test (*Bottom* panel) over all the *N*_*R*_ randomizations, for the pair $(I,D)$ and the threshold ${Q}_{300}$ at Pontelagoscuro, corresponding to the following data sets: the Observations–Control (*Obs*, *Ctrl*), the Observations–RCP4.5 (*Obs*, *RCP45*), and the Control–RCP4.5 (*Ctrl*, *RCP45*). The *dashed* horizontal line corresponds to the 5% reference level. The *vertical* labels indicate possible change-point years. Also reported are the sample sizes (in parentheses). The labels “F:I”, “F:D”, “F:(I, D)”, and “C:(I, D)” indicate, respectively, the distributions ${F}_{I}$, ${F}_{D}$, ${{\bf{F}}}_{ID}$, and the copula ${{\bf{C}}}_{ID}$.

Similar conclusions can be drawn considering the plots in Fig. 9(*Top* panel), concerning the pairs $(I,D)$'s describing the drought episodes, and all the distributions of interest. Again, at a 5% level, no change-points are present in the observed data, as well as in the data simulated during the control period. Instead, around the period 2044–2045, both ${F}_{I}$ and ${F}_{D}$ (and, hence, also ${{\bf{F}}}_{ID}$) show changes, whereas the copula ${{\bf{C}}}_{ID}$ seems to be unaltered: this represents a feature of the models, and may (or may not) be desirable when the IPCC RCP4.5 scenario is considered.

Finally, thanks to the Sklar’s Theorem representation, by combining the results of univariate homogeneity tests and those of suitable (non-parametric) Copula-equality tests, it is possible to understand which distributional (probabilistic) components are (or are not) affected. In particular, the results shown in Fig. 9(*Bottom* panel), concerning the pair (*I*, *D*), indicate that, at a 5% level, the copula of the Observations is different from the ones of the data simulated during the control period and the future temporal horizon, whereas these latter two datasets apparently show the same dependence structure. In turn, the climate-hydrology model is unable to reproduce the copula of the observed data over the control period, while it replicates the one of the Control data over the future temporal horizon: this may represent a flaw of the models.

It is worth stressing that the same analyses cannot be carried out for the variables $P,T,Q$: the reason is that the available sample sizes are not large enough, and the Copula-equality tests may lack of power, yielding results of little statistical relevance (instead, the Change-Point tests are robust against small sample sizes).

## Summary and Conclusions

In this work, a thorough investigation of the behavior and the performances of linked climate-hydrology models is carried out. On the one hand, innovative analysis paradigms, involving the probability distributions of the data, are exploited. On the other hand, making full use of the Sklar’s representation of joint probability distributions, a multivariate approach is proposed, where both the univariate marginals and the relevant dependence structures (i.e., the copulas) can be considered altogether.

Suitable (non-parametric) statistical tests are used to check a number of features and assumptions of utmost importance in climate-hydrology studies, especially considering the investigation of climate changes. In particular, the following issues are illustrated and discussed:

- whether the model is able to reproduce the distributions of the data over which it has been calibrated, both univariate and multivariate (via homogeneity and Copula-equality tests);
- to what extent the model is able to reproduce (or alter) the degree of association between different non-independent variables (via Kendall and Spearman tests);
- whether the model is able to introduce change-points in the probability laws of interest (via Change-Point tests), which represent the fingerprints of possible climate changes;

- possible failures of the statistical tools adopted.

The analyses proposed exploit recent and innovative statistical tests: used in combination with traditional validation techniques, they may help in improving the assessment of climate-hydrology models, and provide important information concerning the behavior (and the flaws) of such models. In turn, also impact/mitigation studies, as well as estimates of the damages induced by (possible) future environmental changes and disasters, may benefit from the thorough and comprehensive additional analyses sketched out in this work. Most importantly, the methodological approach outlined here is appropriate also in contexts different from climate-hydrology studies, in order to evaluate the performance of *any* model of interest: in particular, methods to check a model *per se* are sketched out, investigating whether its outcomes are (statistically) consistent.

## Electronic supplementary material

Supplementary Material
