# Supplementary material for: A distributional multivariate approach for assessing performance of climate-hydrology models
Source: Sci Rep. 2017 Sep 21;7:12071. doi: 10.1038/s41598-017-12343-1 (PMC5608904; doi:10.1038/s41598-017-12343-1)
Supplement: Supplementary file 1 — Supplementary Material [file 41598_2017_12343_MOESM1_ESM.pdf]

A distributional multivariate approach for assessing performance of climate-hydrology models

SUPPLEMENTARY MATERIAL

Nature Scientific Reports

R. Vezzoli
Centro Euro-Mediterraneo sui Cambiamenti Climatici (CMCC)
Regional Models and geo-Hydrological Impacts Division (REMHI)
Capua (CE), I-81043, Italy
renata.vezzoli@cmcc.it

G. Salvadori*
Università del Salento
Dipartimento di Matematica e Fisica
Lecce, I-73100, Italy
gianfausto.salvadori@unisalento.it

C. De Michele*
Politecnico di Milano
Dipartimento di Ingegneria Civile ed Ambientale (DICA)
Milano, I-20133, Italy
carlo.demichele@polimi.it

July 27, 2017

*Corresponding Author.

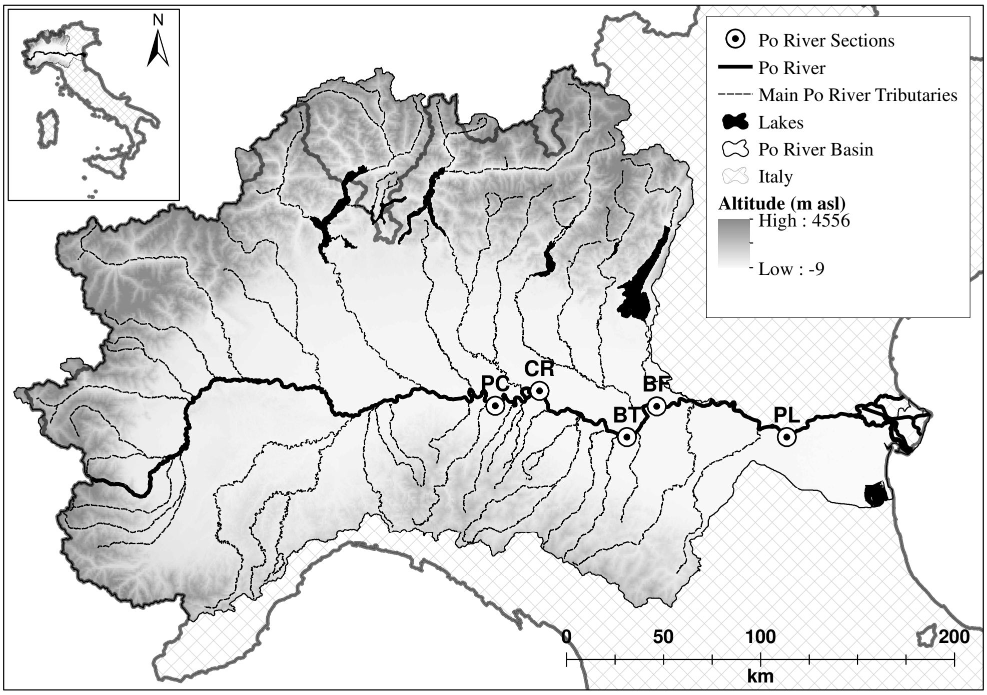

Figure 1: The Po river basin and the five sections of interest: from East to West, Piacenza (PC), Cremona (CR), Boretto (BT), Borgoforte (BF), and Pontelagoscuro (PL). The plot is generated using ArcGIS 10.2.2 - Desktop Version 10.2.2.3552 (`http://www.esri.com`).

**Station**	**Observed**	N_{Obs}	**Control**	N_{Ctrl}	N_{RCP45}
Piacenza	446.1	58	468.0	49	217
Cremona	516.7	64	534.9	54	220
Boretto	549.4	64	566.2	48	202
Borgoforte	647.0	64	667.5	44	197
Pontelagoscuro	778.9	67	715.2	43	196

Table 1: Values of the thresholds Q_{300} (in m^3/s) for the control period 1982–2005, for the five river sections of interest, for both the observed (*Obs*) and the control (*Ctrl*) time series — see text. Also shown are the sizes N of the samples of droughts extracted from the corresponding data bases. The right-most column reports the sizes of the samples extracted from the period 2006-2100 (*RCP45*) using the same thresholds as of the control period.

Climate-hydrology analysis (univariate): variables P, T, Q

Kolmogorov-Smirnov (KS) and Anderson-Darling (AD) homogeneity tests

In this Section, a univariate statistical analysis of the variables P, T, and Q will be presented, for all the five available river sections (viz., Boretto, Borgoforte, Cremona, Piacenza, and Pontelagoscuro): details can be found in the paper.

Each sub-section contains the following plots related to a single station, as indicated in the corresponding sub-section title. Here the non-parametric Kolmogorov-Smirnov (KS) and Anderson-Darling (AD) homogeneity tests are used to check whether the Null hypothesis "$\mathcal{H}_0$: the (univariate) samples come from the same distribution" should be rejected. Note that the KS test is more powerful concerning the body of the (unknown) distribution, while the AD one is more specific for the tails. Shown are the p-Values of the tests. The *dashed* horizontal line corresponds to the 5% reference level.

Top panel. Plotted are the p-Values of the KS (*white* bars) and AD (*grey* bars) homogeneity tests, for the variable P, corresponding to the following pairs of data sets: the Observations–Control (*Obs,Ctrl*), the Observations–RCP4.5 (*Obs,RCP45*), and the Control–RCP4.5 (*Ctrl,RCP45*).

Middle panel. Same as *top panel*, for the variable T.

Bottom panel. Same as *top panel*, for the variable Q.

Boretto: KS and AD tests

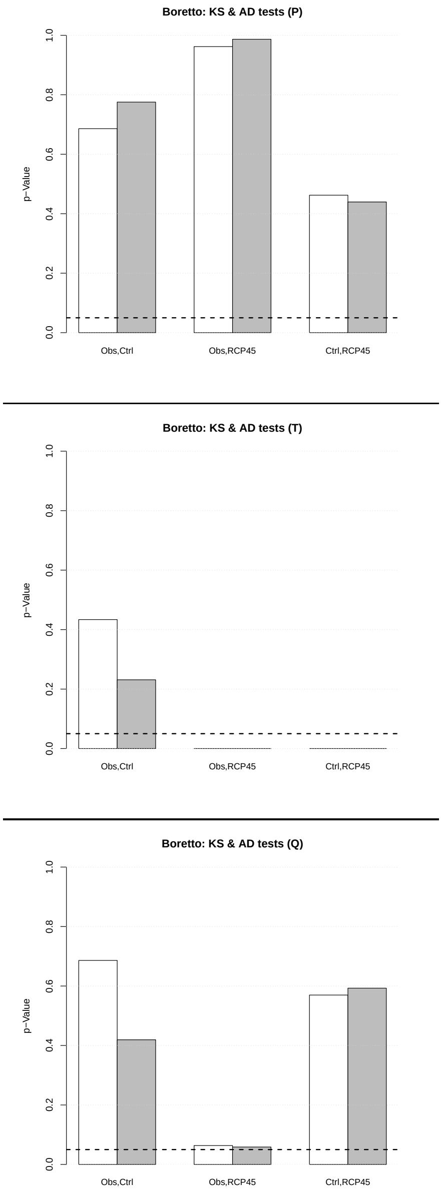

Figure 2: see text for explanation.

Borgoforte: KS and AD tests

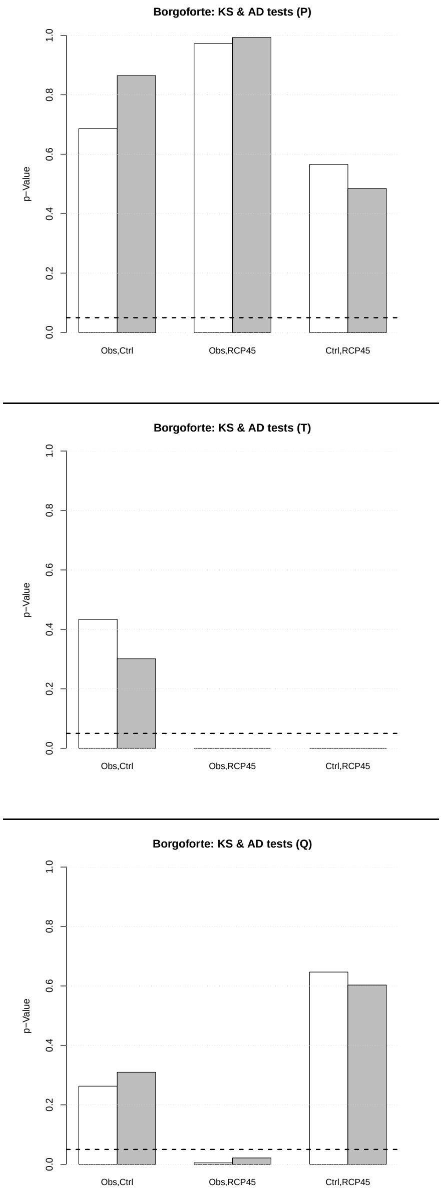

Figure 3: see text for explanation.

Cremona: KS and AD tests

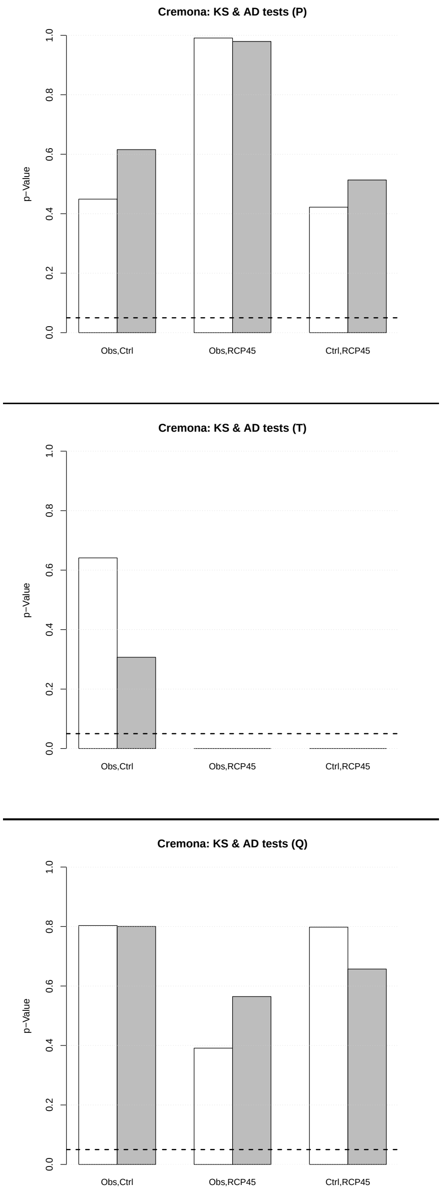

Figure 4: see text for explanation.

Piacenza: KS and AD tests

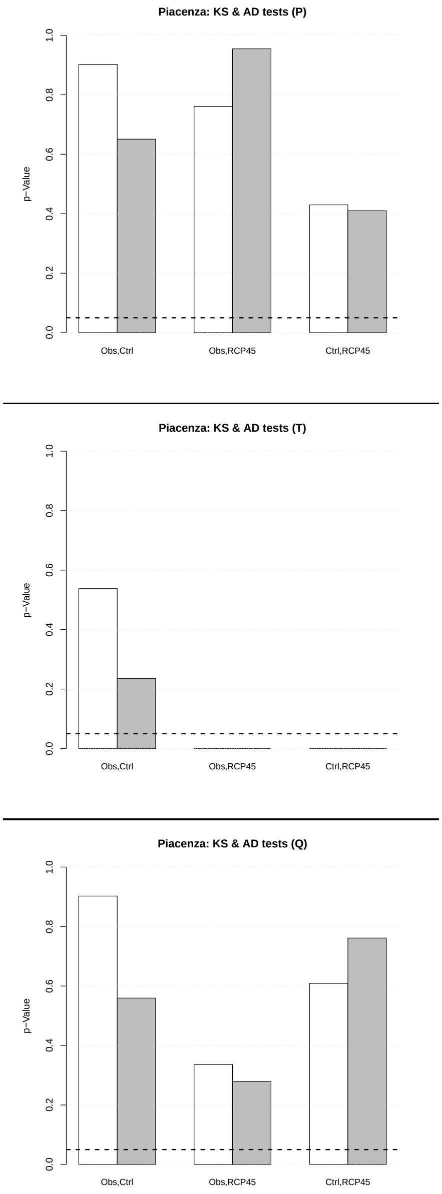

Figure 5: see text for explanation.

Pontelagoscuro: KS and AD tests

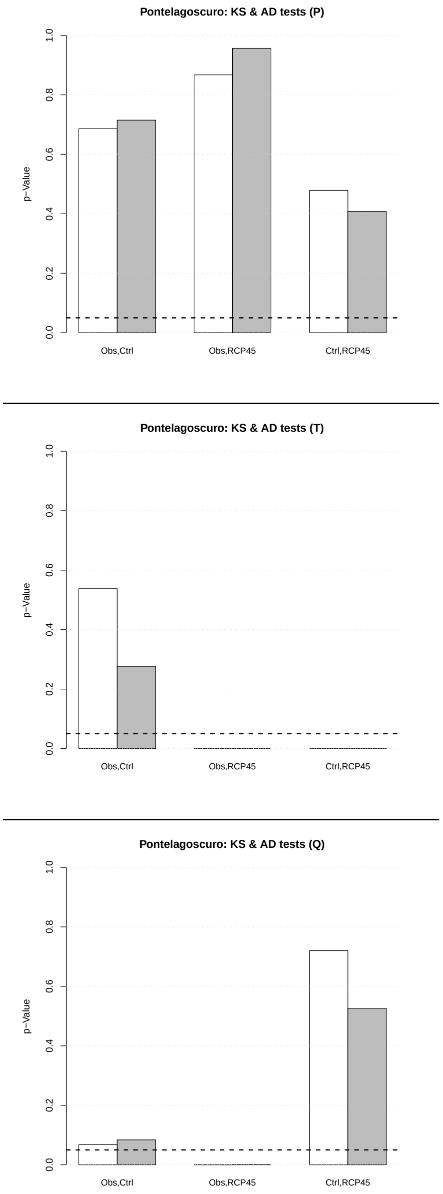

Figure 6: see text for explanation.

Climate-hydrology analysis (bivariate): variables (P, T, Q)

Kendall τ and Spearman ρ

In this Section, a bivariate statistical analysis of the variables (P, T, Q) will be presented, for all the five available river sections (viz., Boretto, Borgoforte, Cremona, Piacenza, and Pontelagoscuro): details can be found in the paper. For each station, the following plots are presented.

Top panel. Plotted are the estimates of the Kendall τ (*white* bars) and the Spearman ρ (*grey* bars), as well as the corresponding p-Values (*star* markers), for the pair (P, T), corresponding to the following data sets: Observations (*Obs*), Control (*Ctrl*), and RCP4.5 (*RCP45*). The *dashed* horizontal line corresponds to the 5% reference level.

Middle panel. Same as *top panel*, for the pair (P, Q).

Bottom panel. Same as *top panel*, for the pair (T, Q).

Boretto: Kendall τ and Spearman ρ

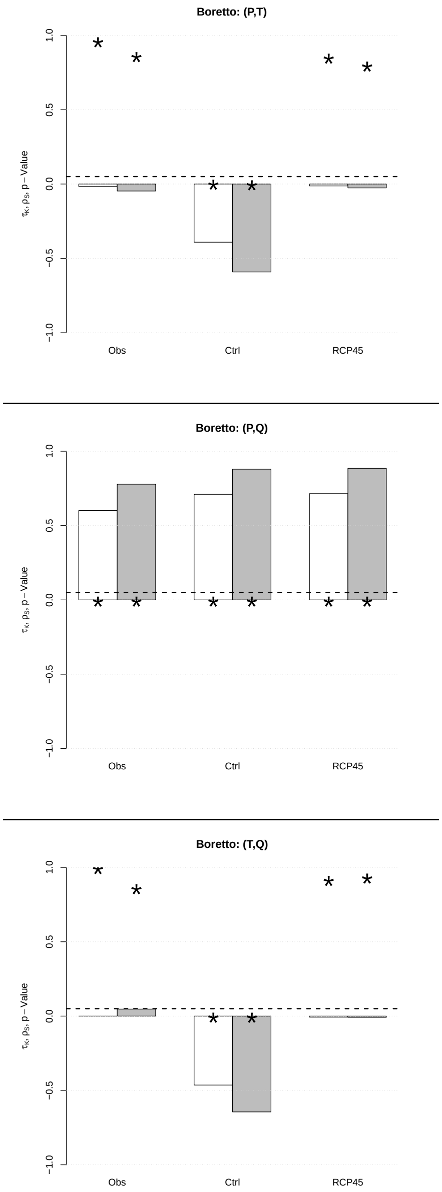

Figure 7: see text for explanation.

Borgoforte: Kendall τ and Spearman ρ

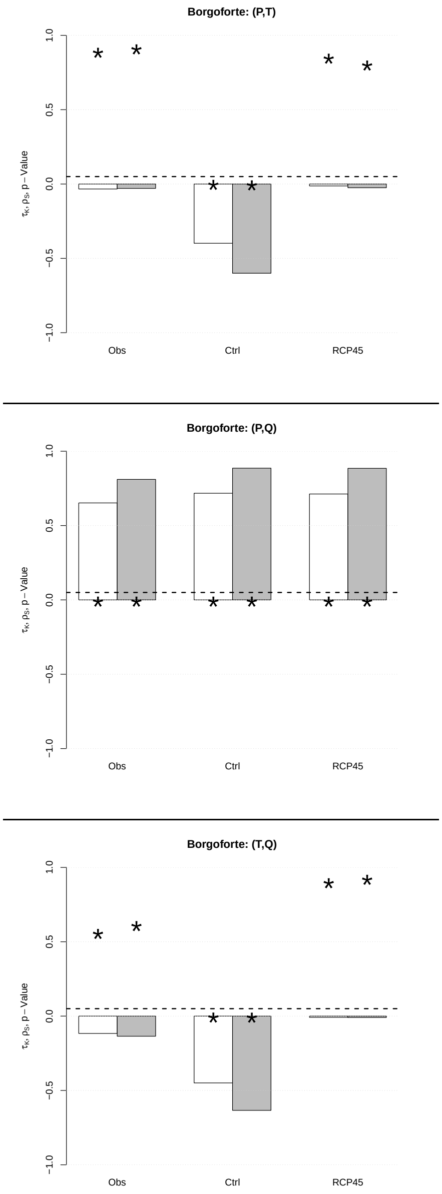

Figure 8: see text for explanation.

Cremona: Kendall τ and Spearman ρ

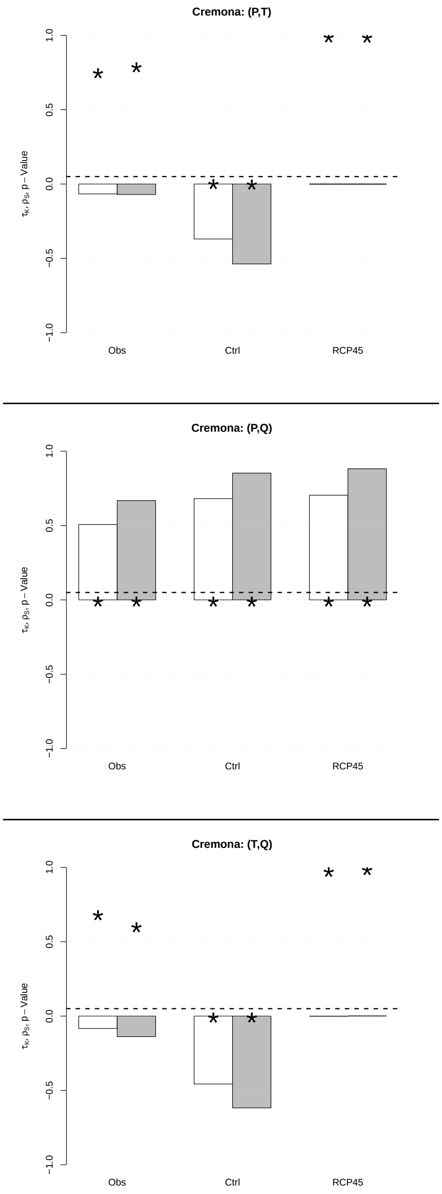

Figure 9: see text for explanation.

Piacenza: Kendall τ and Spearman ρ

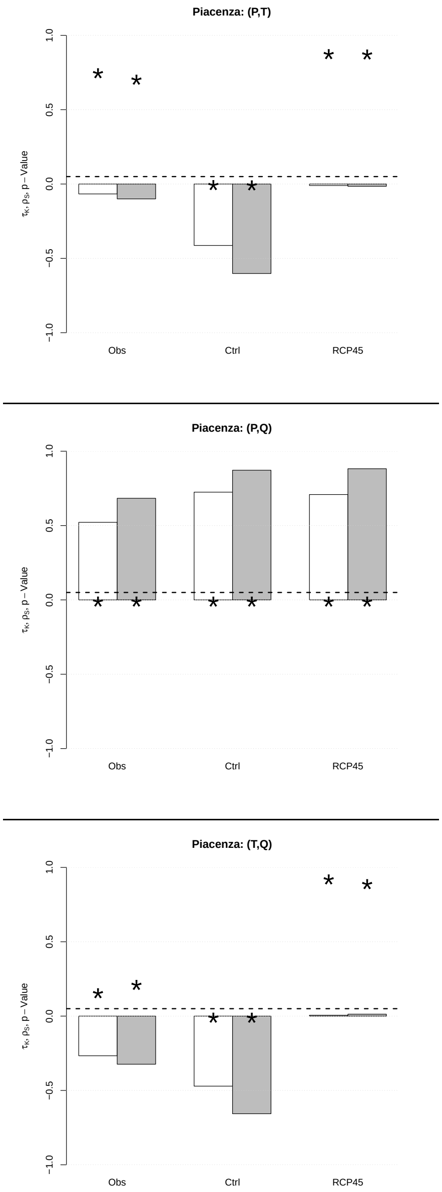

Figure 10: see text for explanation.

Pontelagoscuro: Kendall τ and Spearman ρ

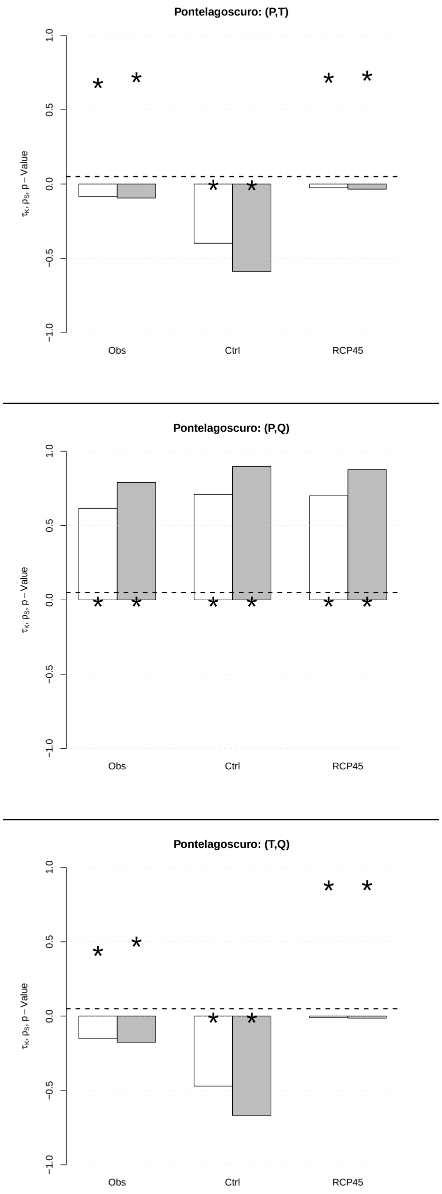

Figure 11: see text for explanation.

Climate-hydrology analysis (multivariate): variables (P, T, Q)

Data, Pseudo-observations, Empirical Copulas, Change-Point tests and Copula-Equality tests

In this Section, a multivariate statistical analysis of the variables (P, T, Q) will be presented, for all the five available river sections (viz., Boretto, Borgoforte, Cremona, Piacenza, and Pontelagoscuro): details can be found in the paper. For each station, the following plots are presented, as indicated in the corresponding sub-section title.

Data, Pseudo-observations and Empirical Copulas. The *left* column concerns the Observations data, the *middle* column concerns the Control data, and the *right* column concerns the RCP4.5 data.

(*Top* row) Plotted are the data of the pairs (P, T), (P, Q), and (T, Q): also reported are the sample sizes.

(*Middle* row) Plotted are the pseudo-observations of the pairs (P, T), (P, Q), and (T, Q): also reported are the sample sizes.

(*Bottom* row) Plotted are the isolines of the Empirical Copulas of the pairs (P, T), (P, Q), and (T, Q), as well as a comparison panel: also reported are the sample sizes.

Change point tests. Shown are the p-Values of the Change-Point tests, corresponding to the following data sets: Observations (*Obs*), Control (*Ctrl*), and RCP4.5 (*RCP45*). From left to right, the distributions considered are: the marginal of the Precipitation P (*F:P*), the marginal of the Temperature T (*F:T*), the marginal of the Discharge Q (*F:Q*), the joint distribution of (P, T) (*F:(P,T)*), the copula of (P, T) (*C:(P,T)*), the joint distribution of (P, Q) (*F:(P,Q)*), the copula of (P, Q) (*C:(P,Q)*), the joint distribution of (T, Q) (*F:(T,Q)*), the copula of (T, Q) (*C:(T,Q)*), the joint distribution of (P, T, Q) (*F:(P,T,Q)*), and the copula of (P, T, Q) (*C:(P,T,Q)*). The *dashed* horizontal line corresponds to the 5% reference level.

Boretto: Data, Pseudo-observations and Empirical Copulas

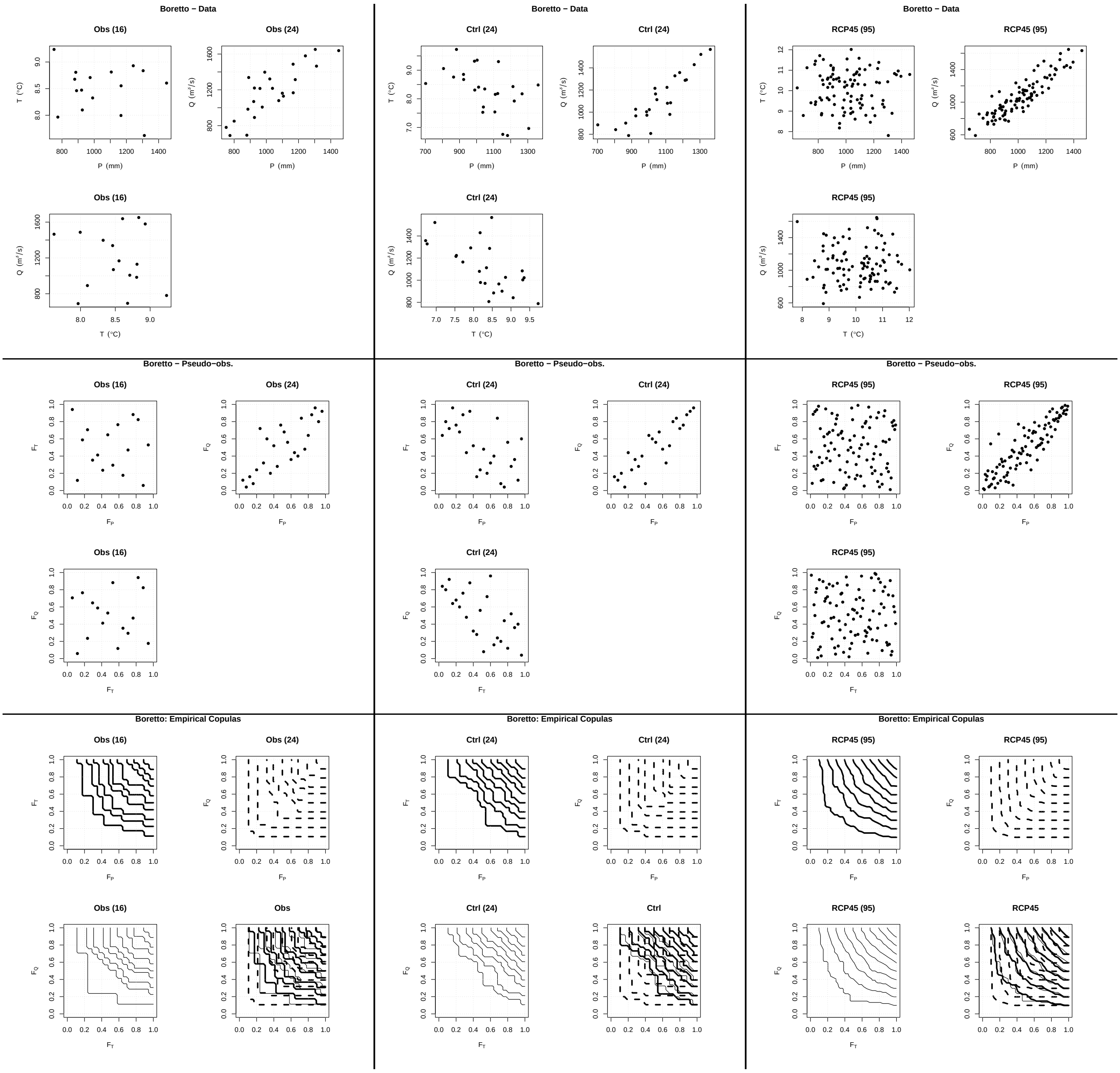

Figure 12: see text for explanation.

Boretto: Change-Point tests

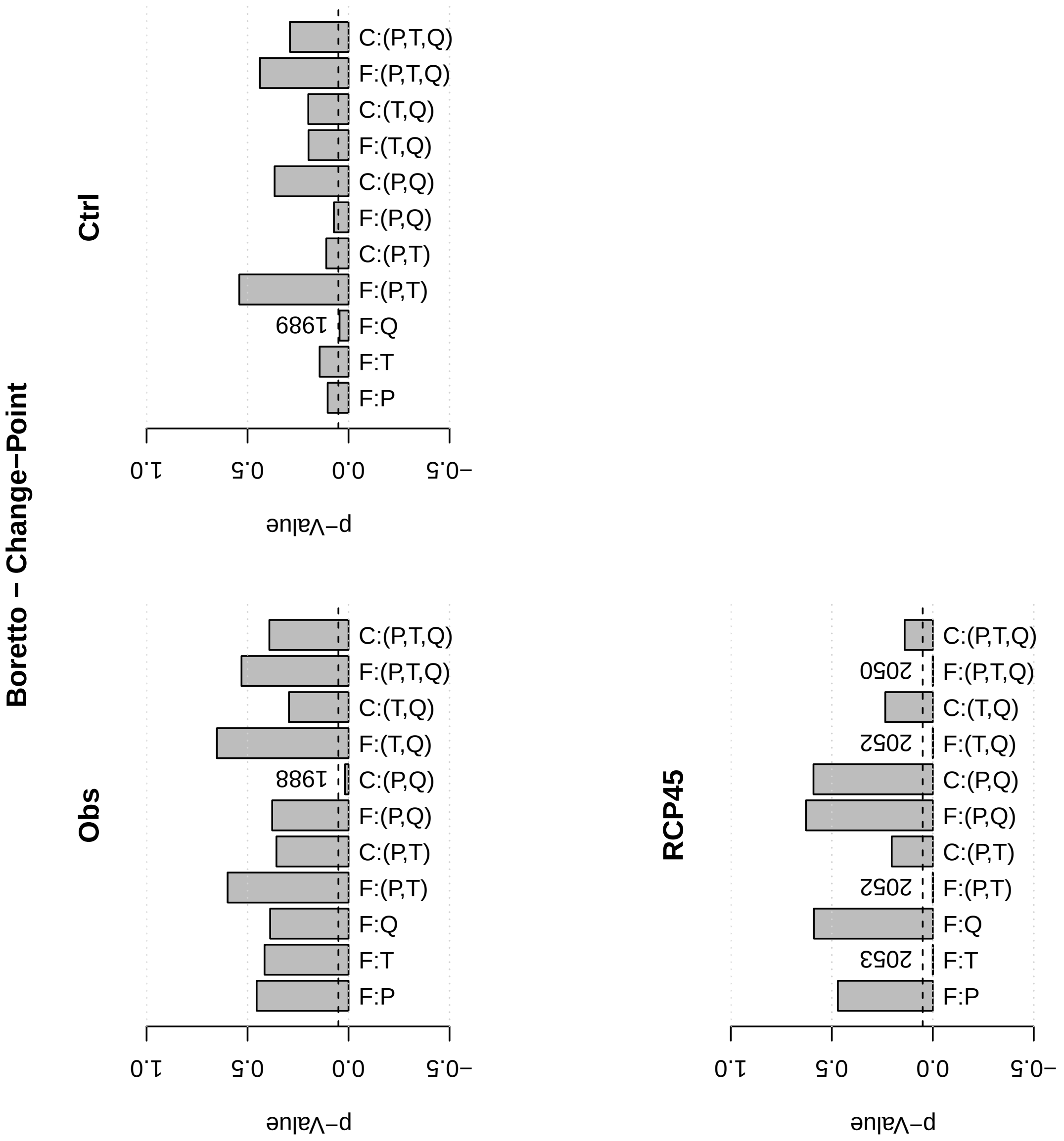

Figure 13: see text for explanation.

Borgoforte: Data, Pseudo-observations and Empirical Copulas

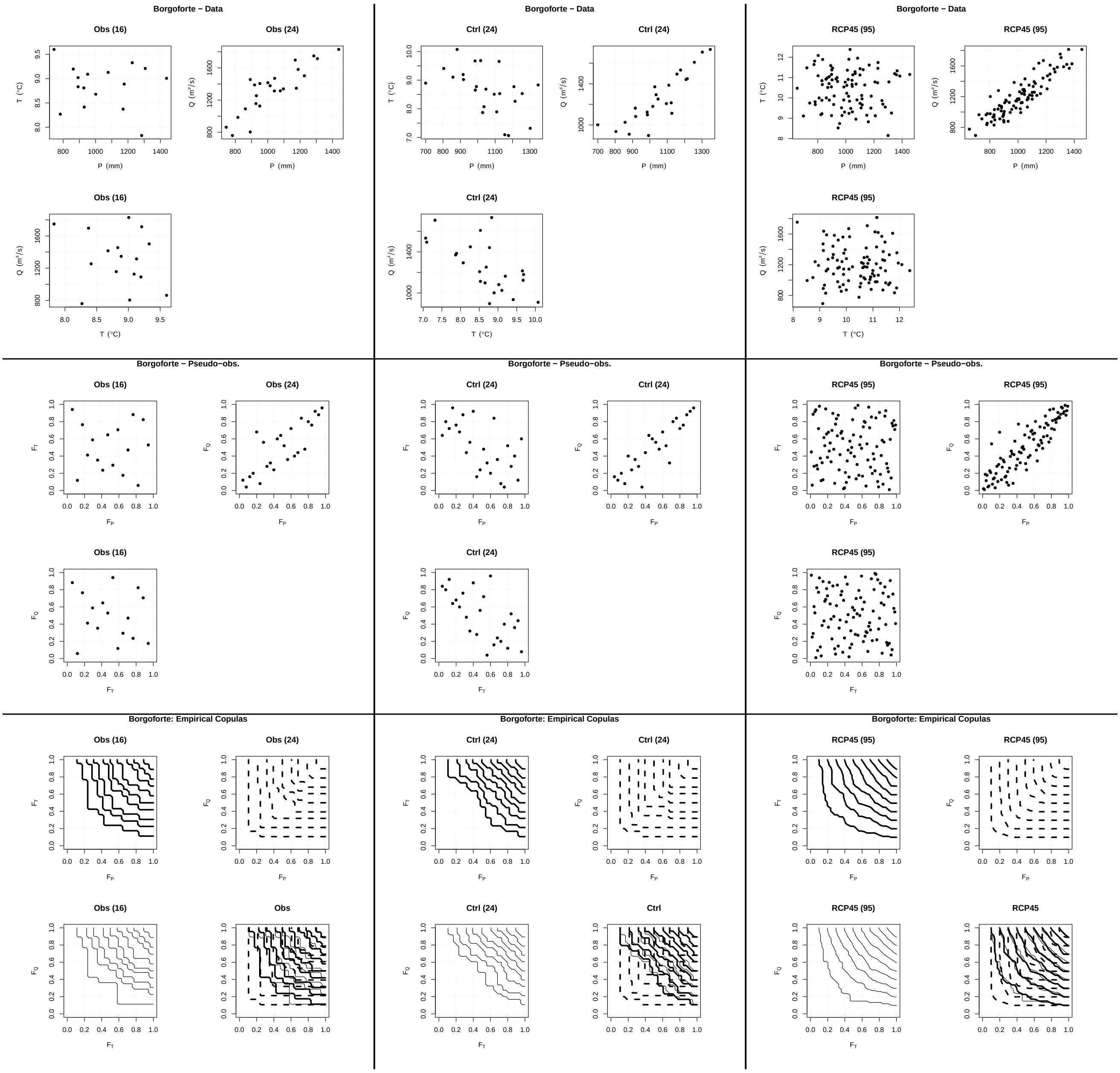

Figure 14: see text for explanation.

Borgoforte: Change-Point tests

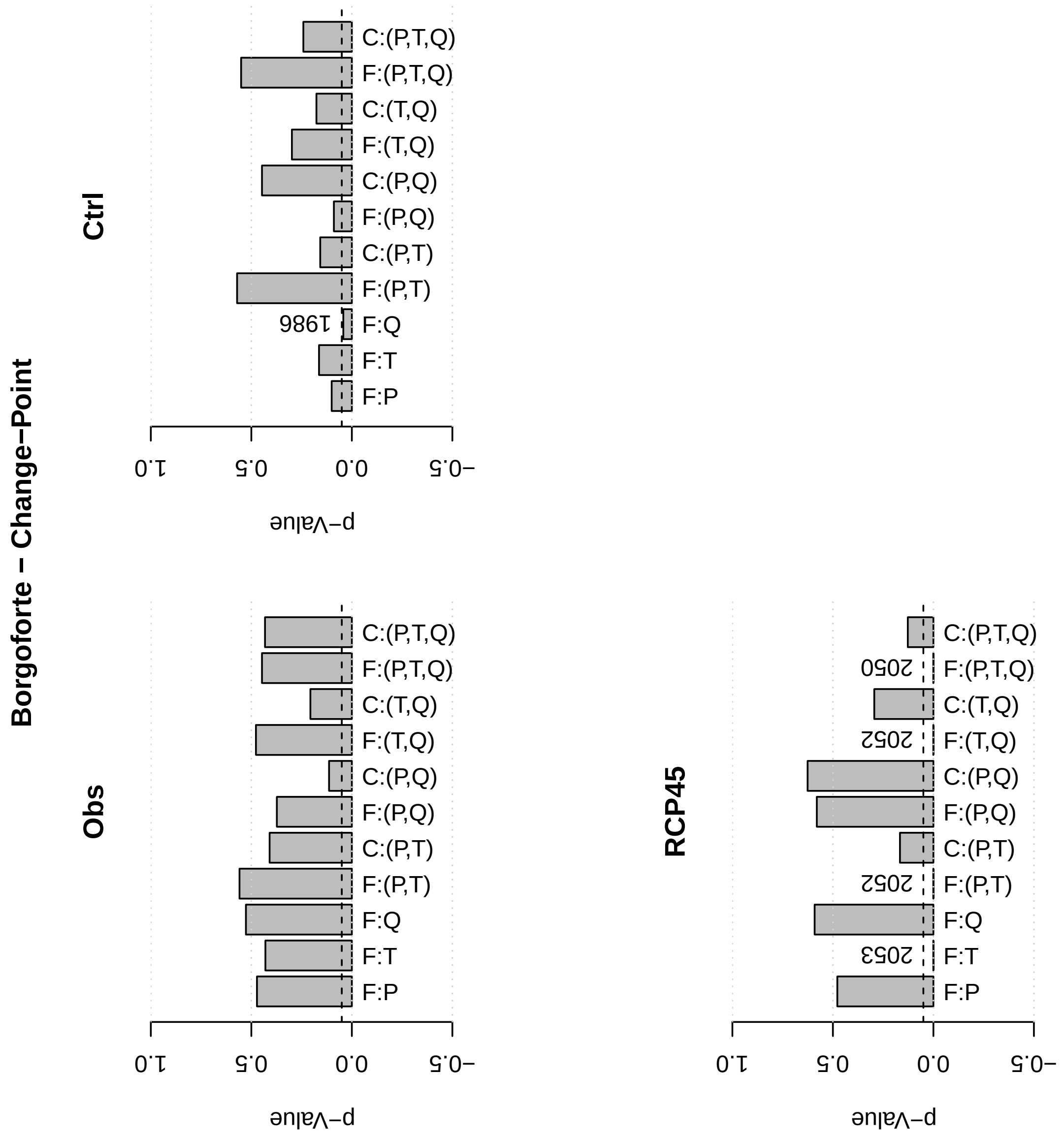

Figure 15: see text for explanation.

Cremona: Data, Pseudo-observations and Empirical Copulas

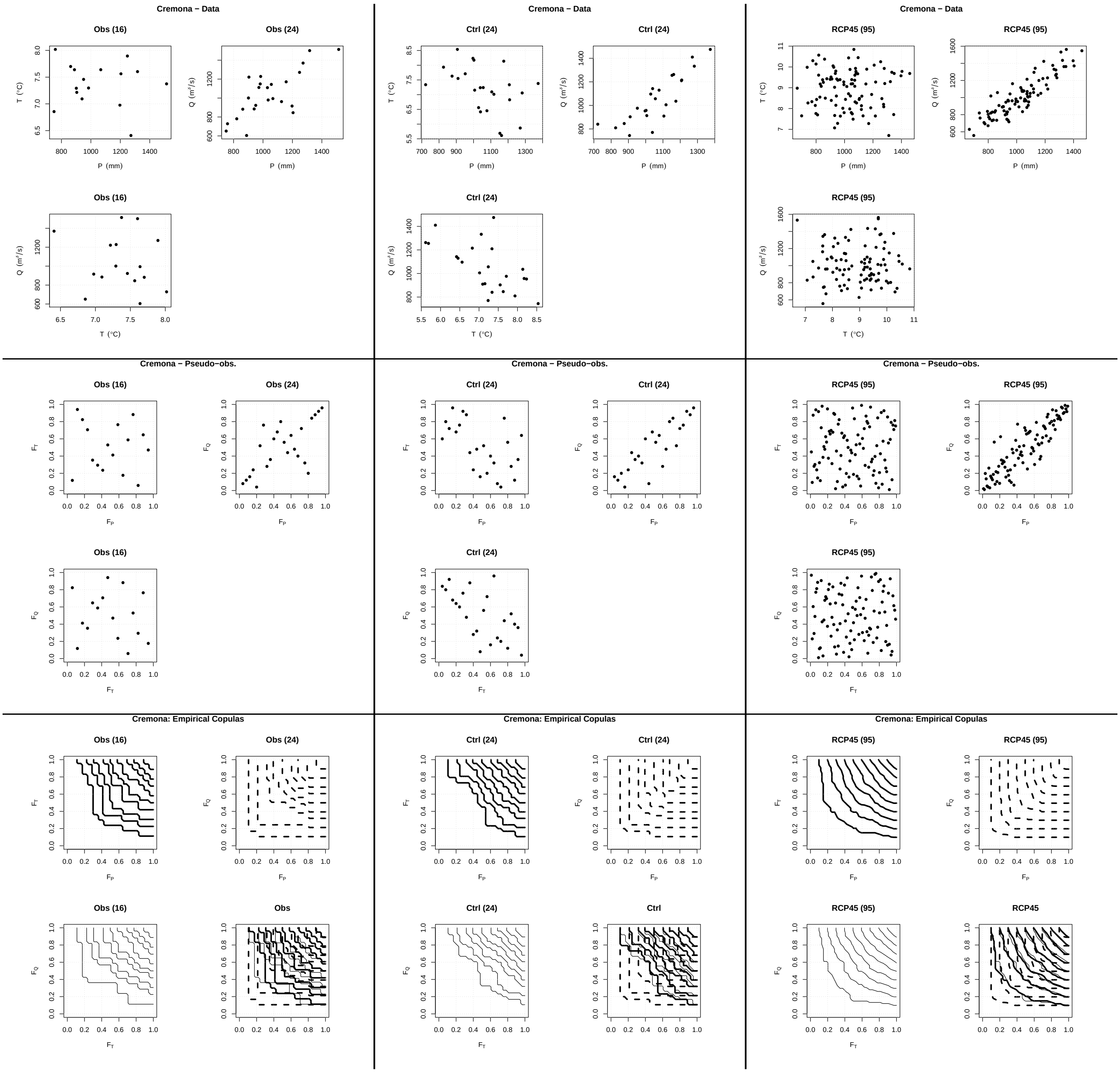

Figure 16: see text for explanation.

Cremona: Change-Point tests

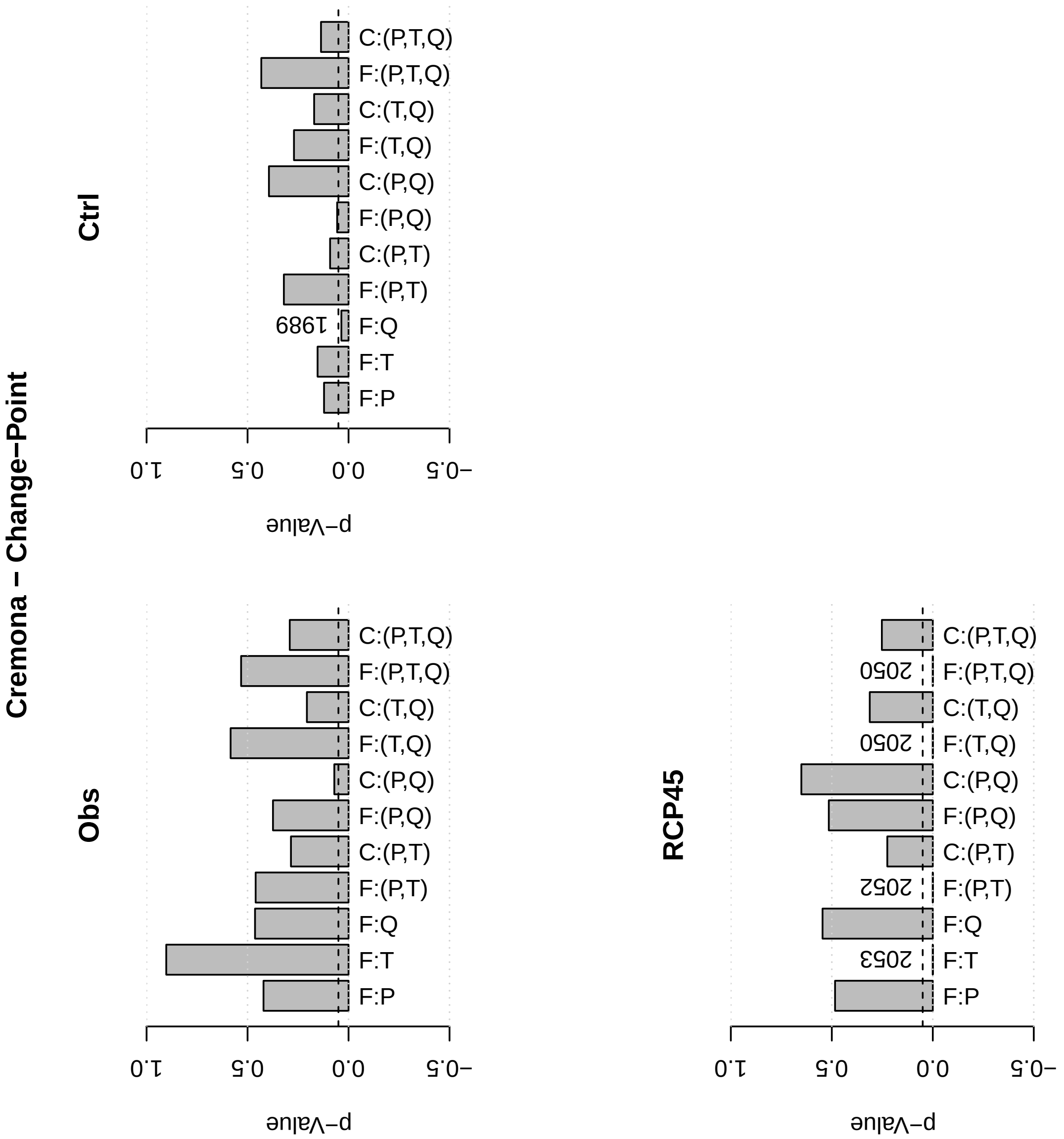

Figure 17: see text for explanation.

Piacenza: Data, Pseudo-observations and Empirical Copulas

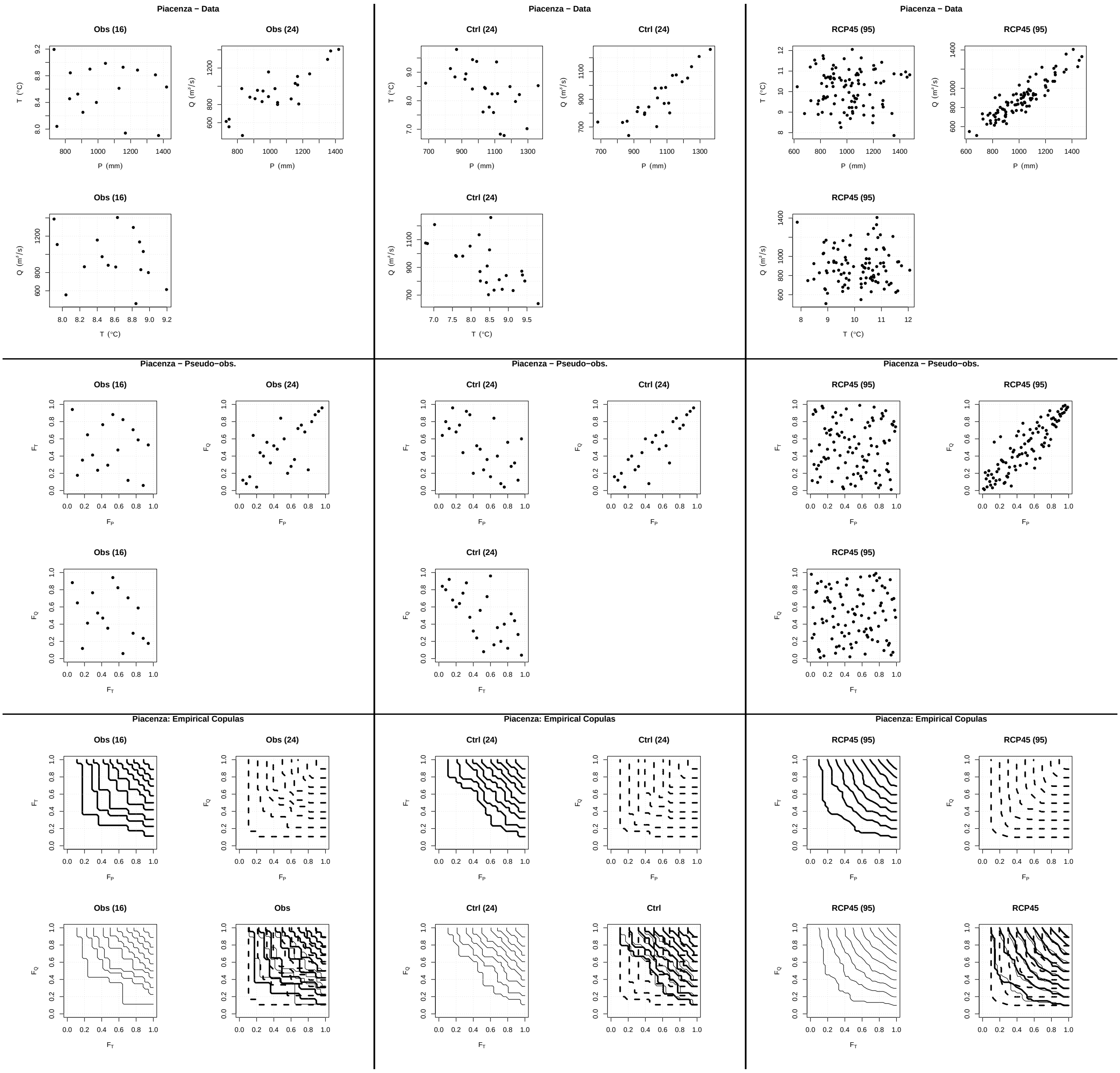

Figure 18: see text for explanation.

Piacenza: Change-Point tests

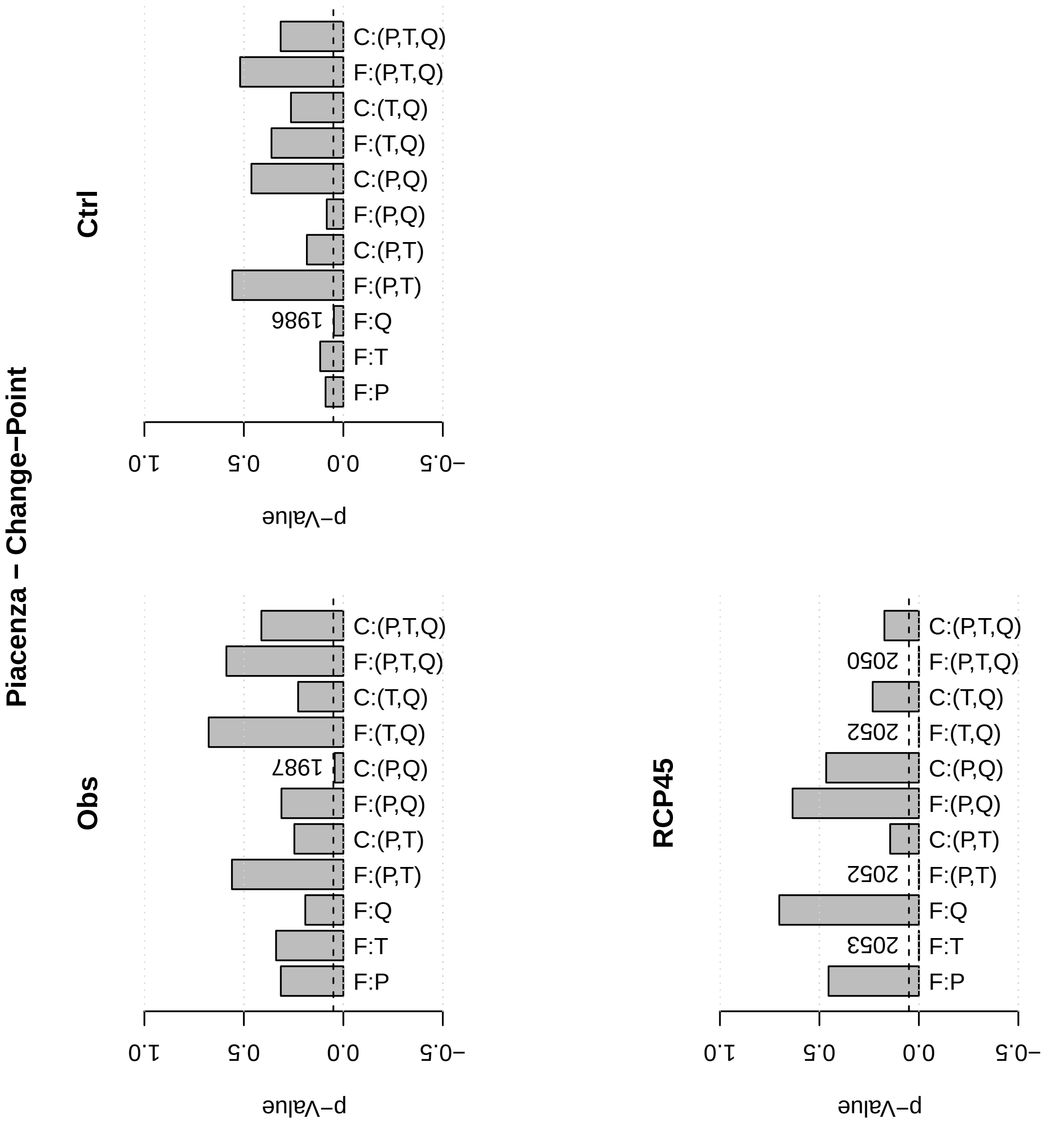

Figure 19: see text for explanation.

Pontelagoscuro: Data, Pseudo-observations and Empirical Copulas

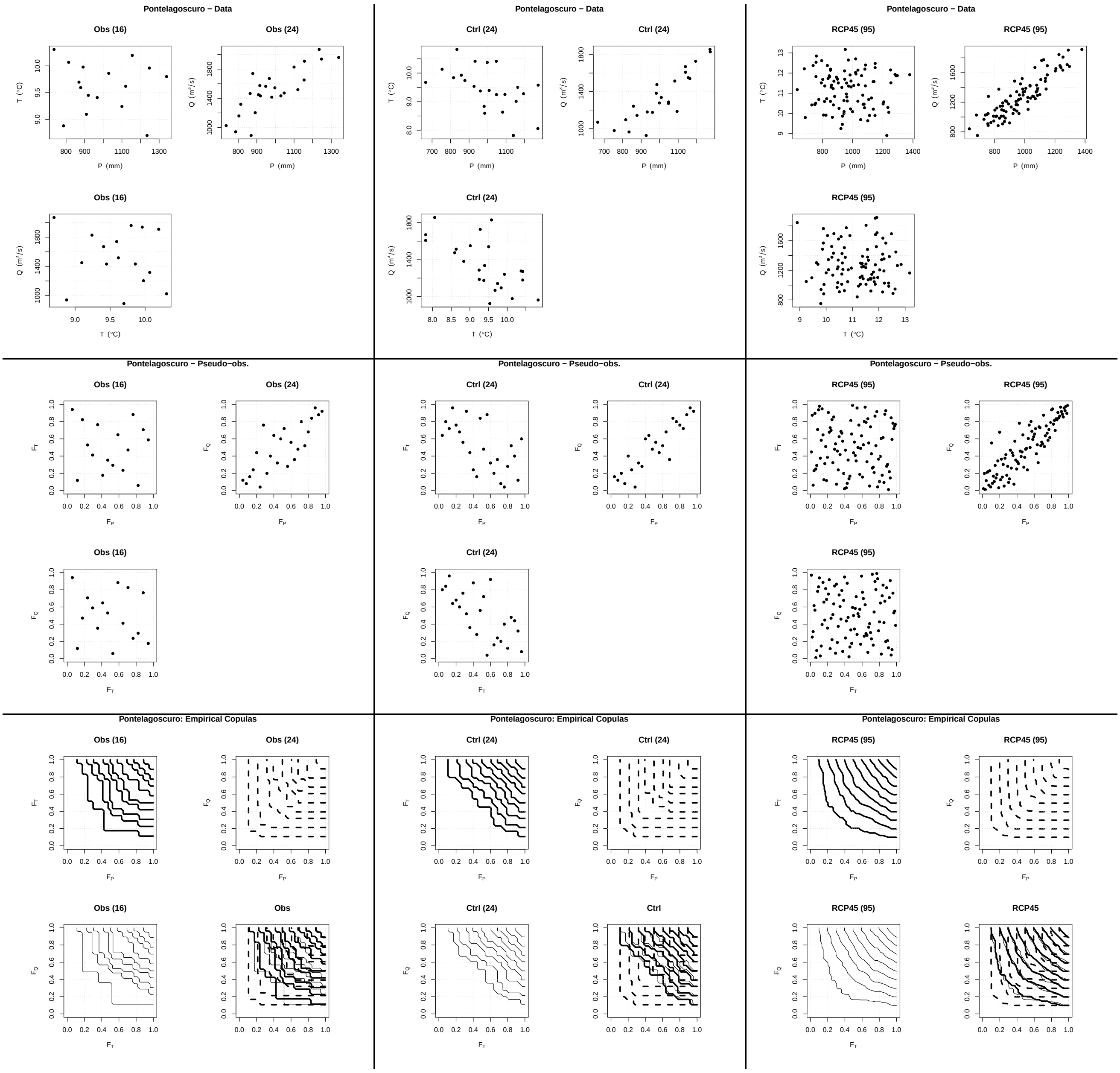

Figure 20: see text for explanation.

Pontelagoscuro: Change-Point tests

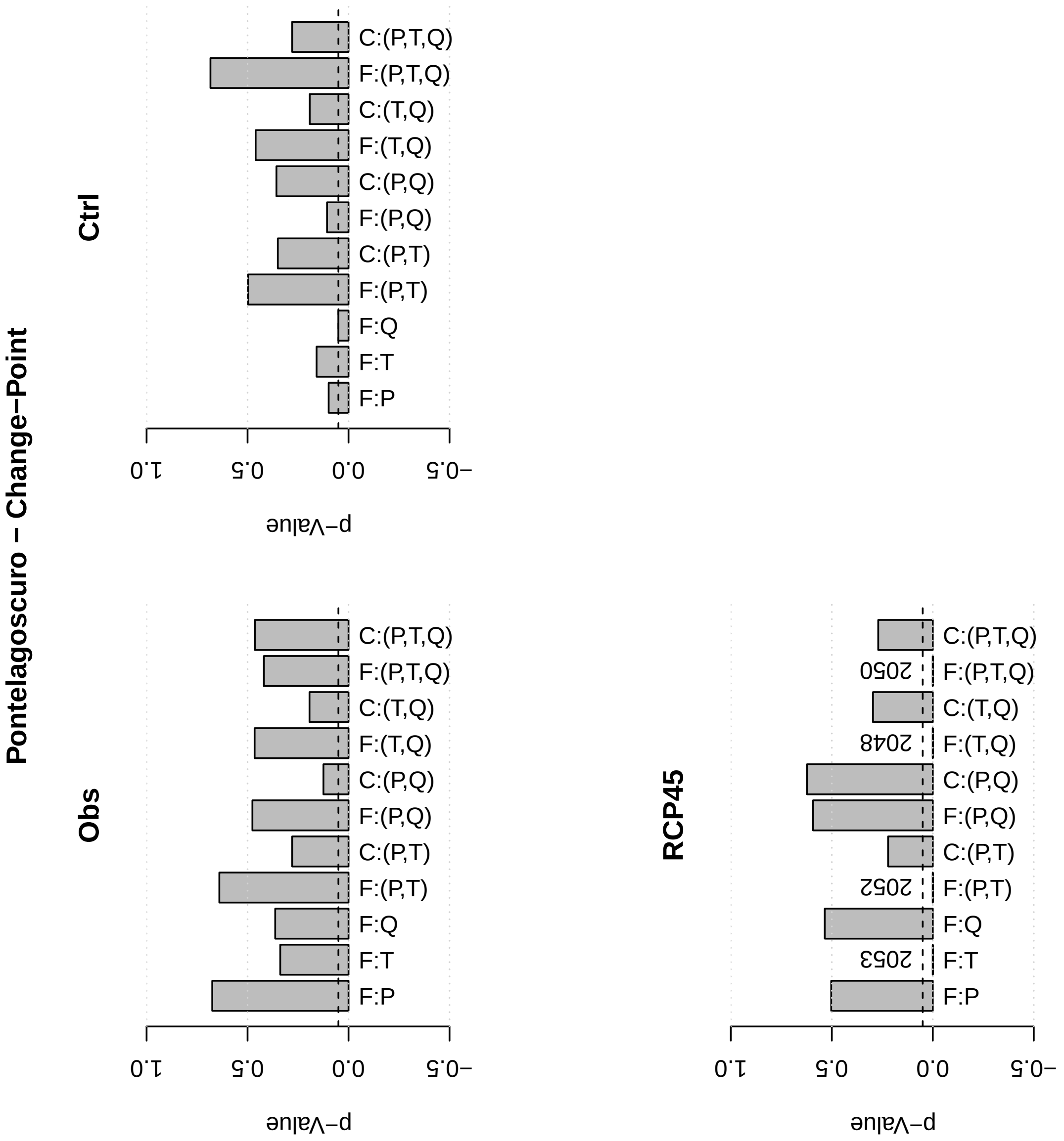

Figure 21: see text for explanation.

Drought analysis (univariate): variables I and D

Kolmogorov-Smirnov (KS) and Anderson-Darling (AD) homogeneity tests

In this Section, a univariate statistical analysis of the drought occurrences will be presented. In particular, all the five available river sections (viz., Boretto, Borgoforte, Cremona, Piacenza, and Pontelagoscuro) will be considered: details can be found in the paper.

Each sub-section contains the following plots related to a single station, as indicated in the corresponding sub-section title. Here, the variables I and D are investigated, considering different pairs of data sets. The non-parametric Kolmogorov-Smirnov (KS) and Anderson-Darling (AD) homogeneity tests are used to check whether the Null hypothesis "$\mathcal{H}_0$: the (univariate) samples come from the same distribution" should be rejected. Note that the KS test is more powerful concerning the body of the (unknown) distribution, while the AD one is more specific for the tails. Shown are the p-Values of the tests: since Ties are present for D, the corresponding box-plots are over all the N_R randomizations. The *dashed* horizontal line corresponds to the 5% reference level.

Plotted are the p-Values of the KS (*white*) and AD (*grey*) tests, for the variables I (*top* panel) and D (*bottom* panel), and the threshold Q_{300}, corresponding to the following pairs of data sets: the Observations–Control (*Obs,Ctrl*), the Observations–RCP4.5 (*Obs,RCP45*), and the Control–RCP4.5 (*Ctrl,RCP45*).

Boretto: KS and AD tests

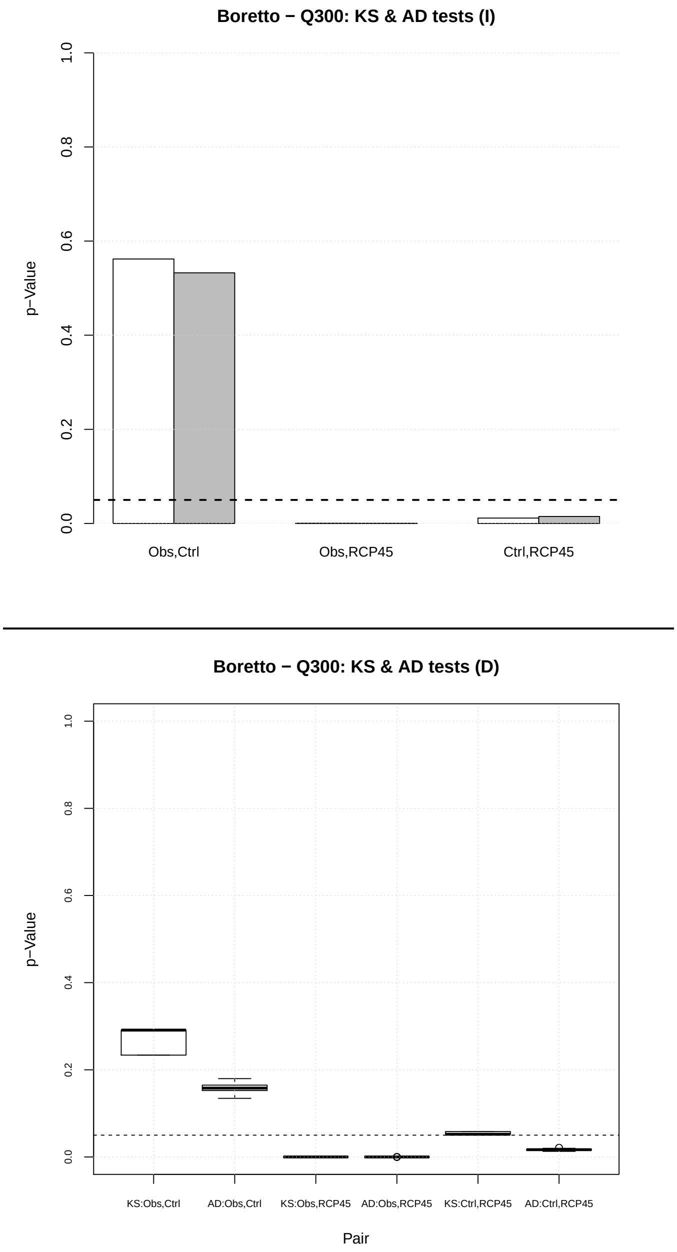

Figure 22: see text for explanation.

Borgoforte: KS and AD tests

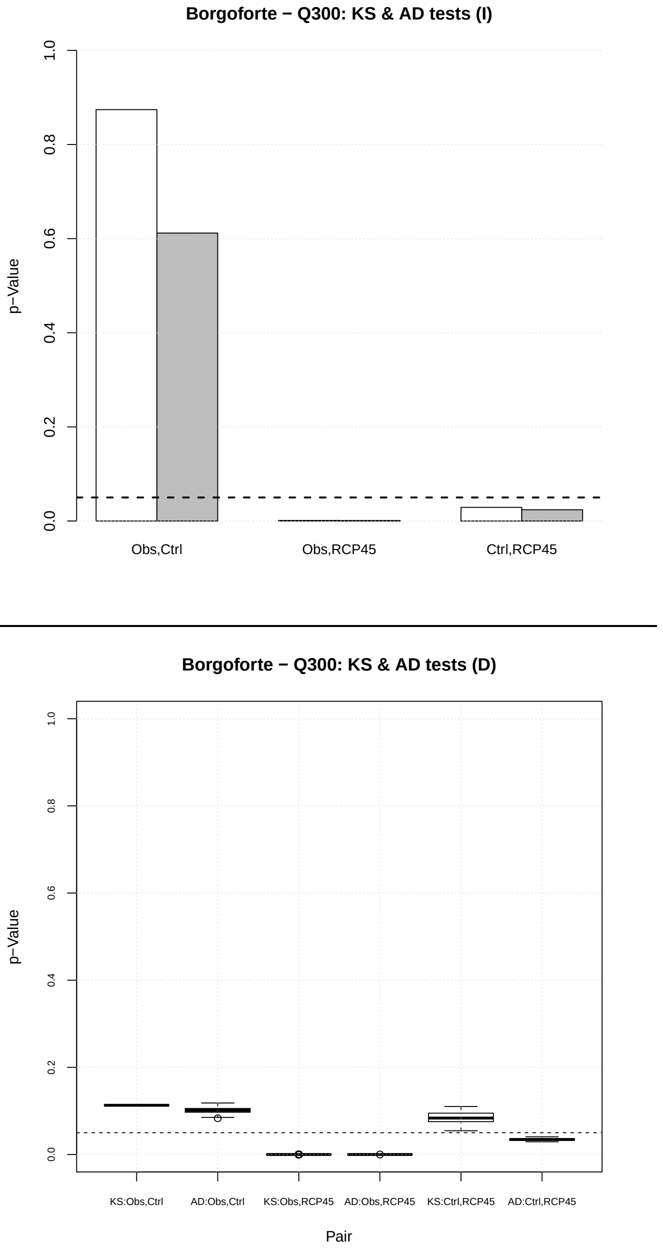

Figure 23: see text for explanation.

Cremona: KS and AD tests

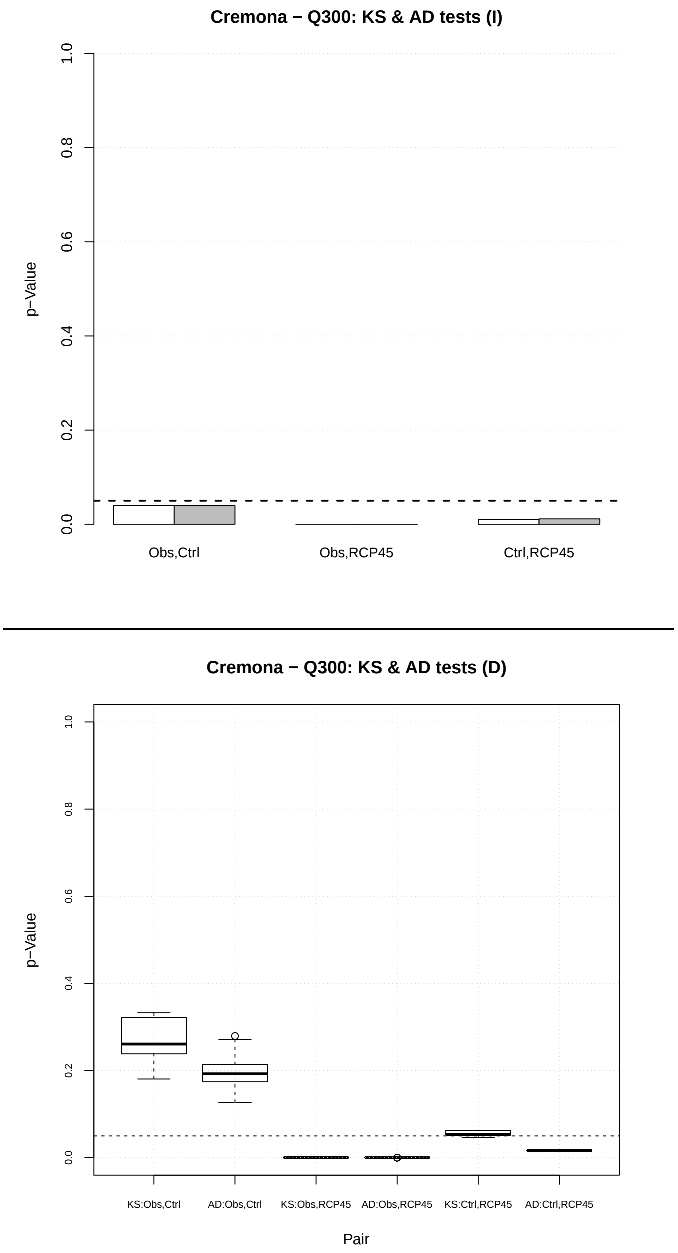

Figure 24: see text for explanation.

Piacenza: KS and AD tests

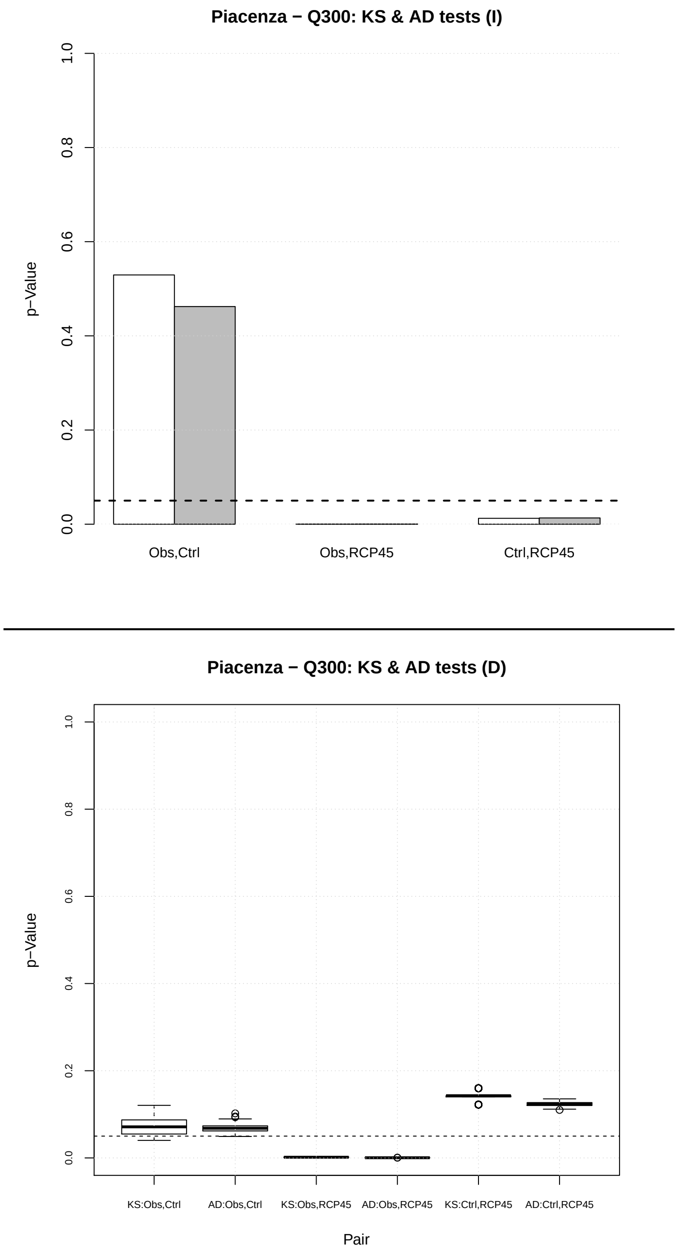

Figure 25: see text for explanation.

Pontelagoscuro: KS and AD tests

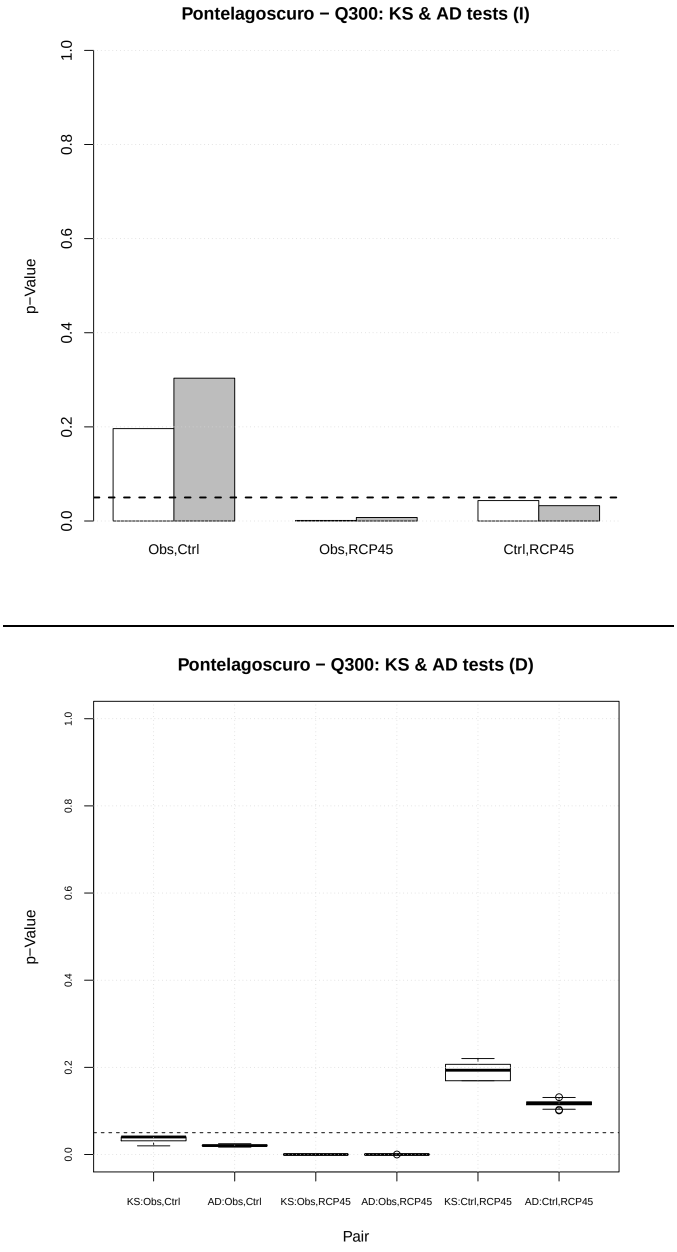

Figure 26: see text for explanation.

Drought analysis (bivariate): variables (I, D)

Kendall τ and Spearman ρ

In this Section, a bivariate statistical analysis of the drought occurrences will be presented. In particular, all the five available river sections (viz., Boretto, Borgoforte, Cremona, Piacenza, and Pontelagoscuro) will be considered: details can be found in the paper. Each sub-section contains the following plots, related to a single station, as indicated in the corresponding sub-section title.

Plotted are the estimates of the Kendall τ (*white* bars) and the Spearman ρ (*grey* bars), as well as the corresponding p-Values (*star* markers), for the pair (I, D) and the threshold Q_{300}, corresponding to the following data sets: Observations (*Obs*), Control (*Ctrl*), and RCP4.5 (*RCP45*). The *dashed* horizontal line corresponds to the 5% reference level.

Boretto: Kendall τ and Spearman ρ

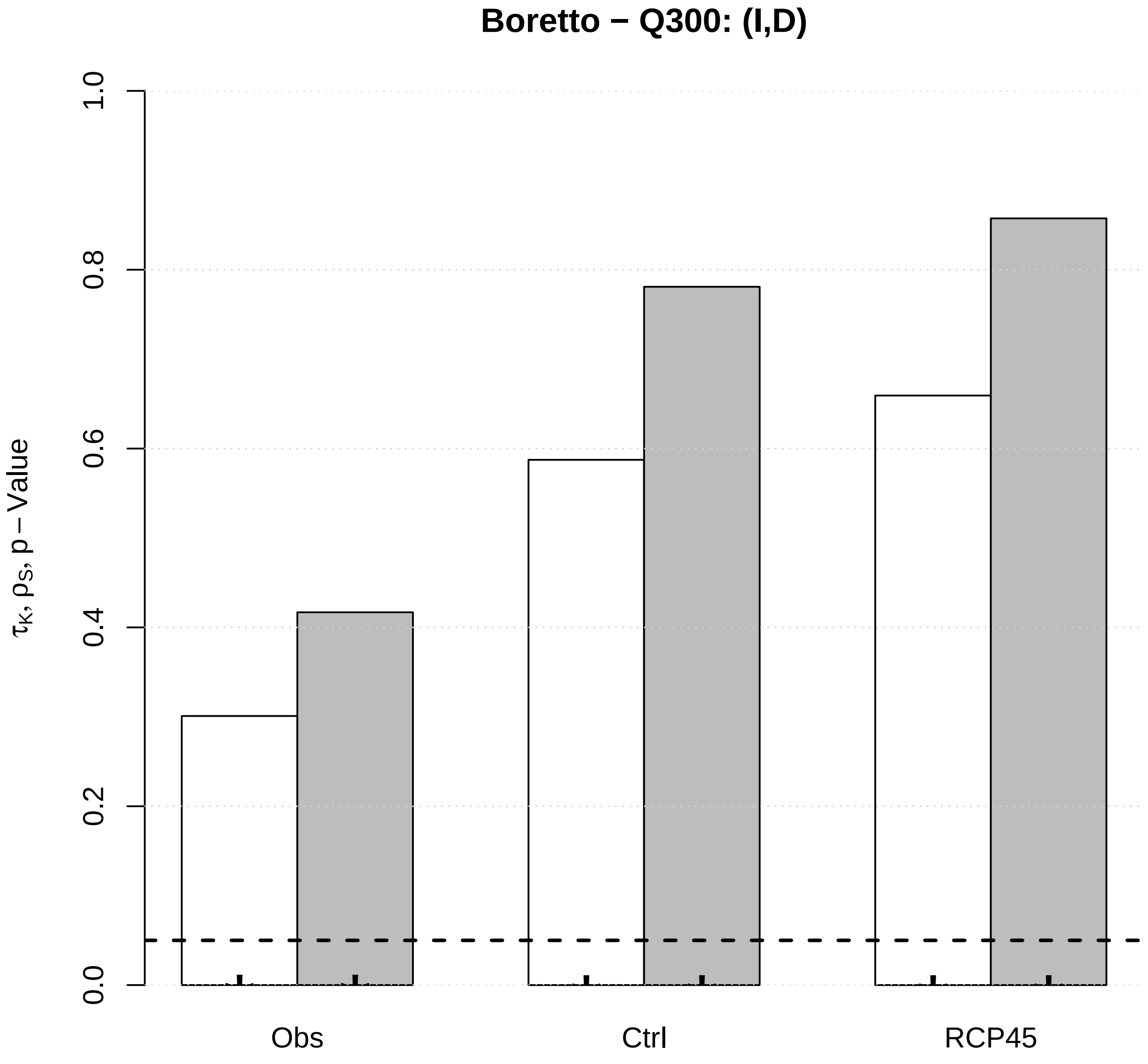

Figure 27: see text for explanation.

Borgoforte: Kendall τ and Spearman ρ

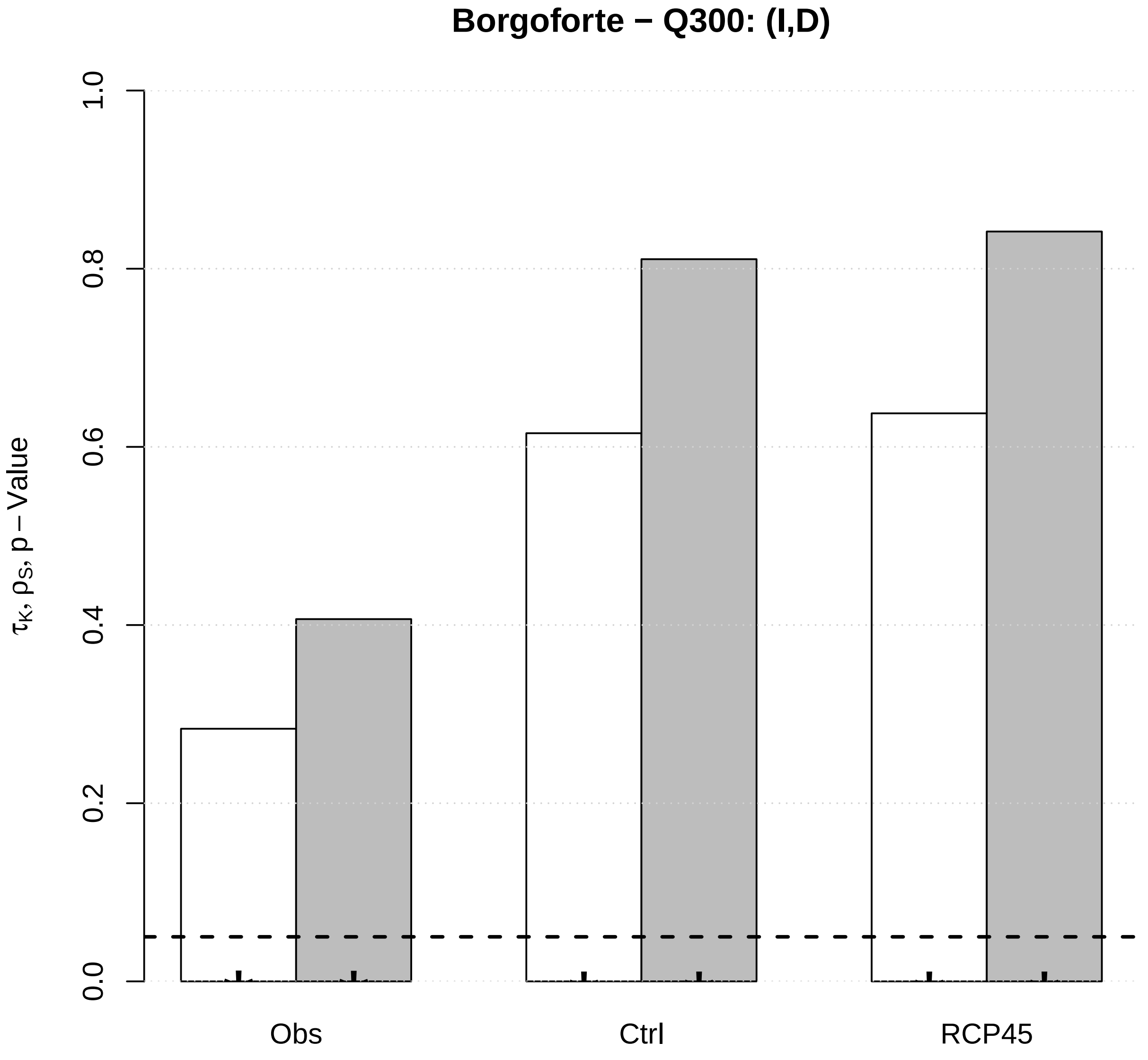

Figure 28: see text for explanation.

Cremona: Kendall τ and Spearman ρ

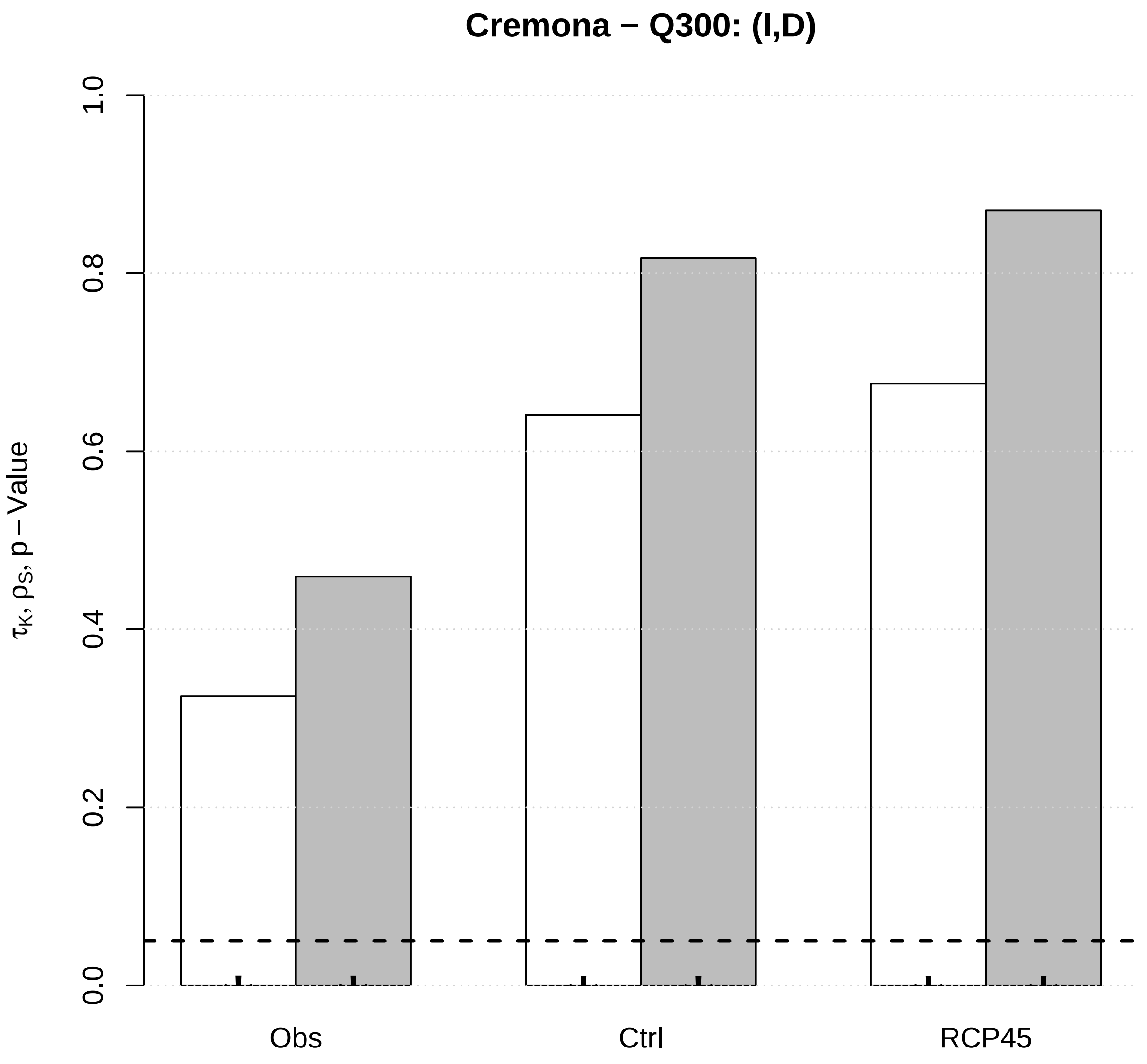

Figure 29: see text for explanation.

Piacenza: Kendall τ and Spearman ρ

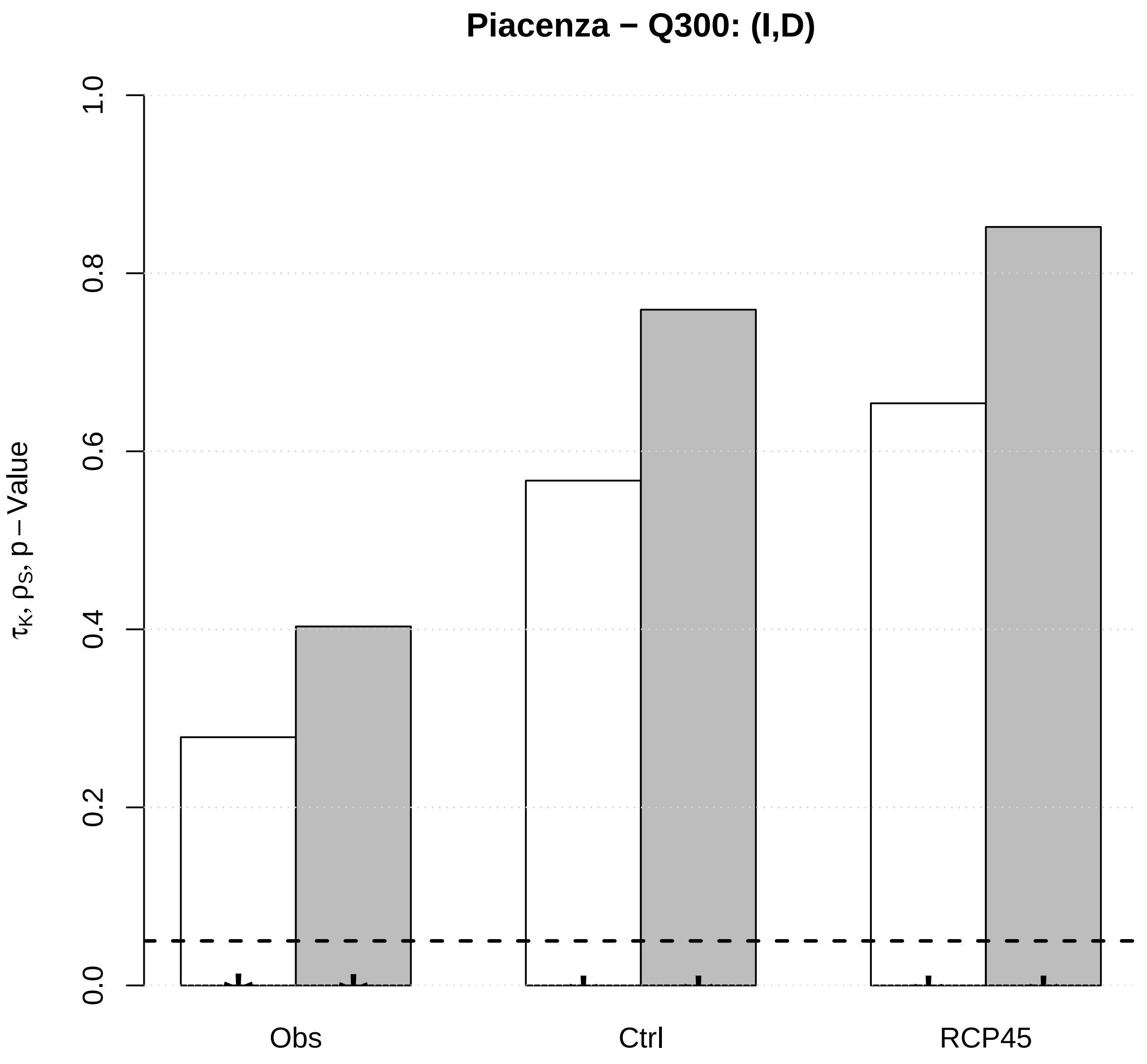

Figure 30: see text for explanation.

Pontelagoscuro: Kendall τ and Spearman ρ

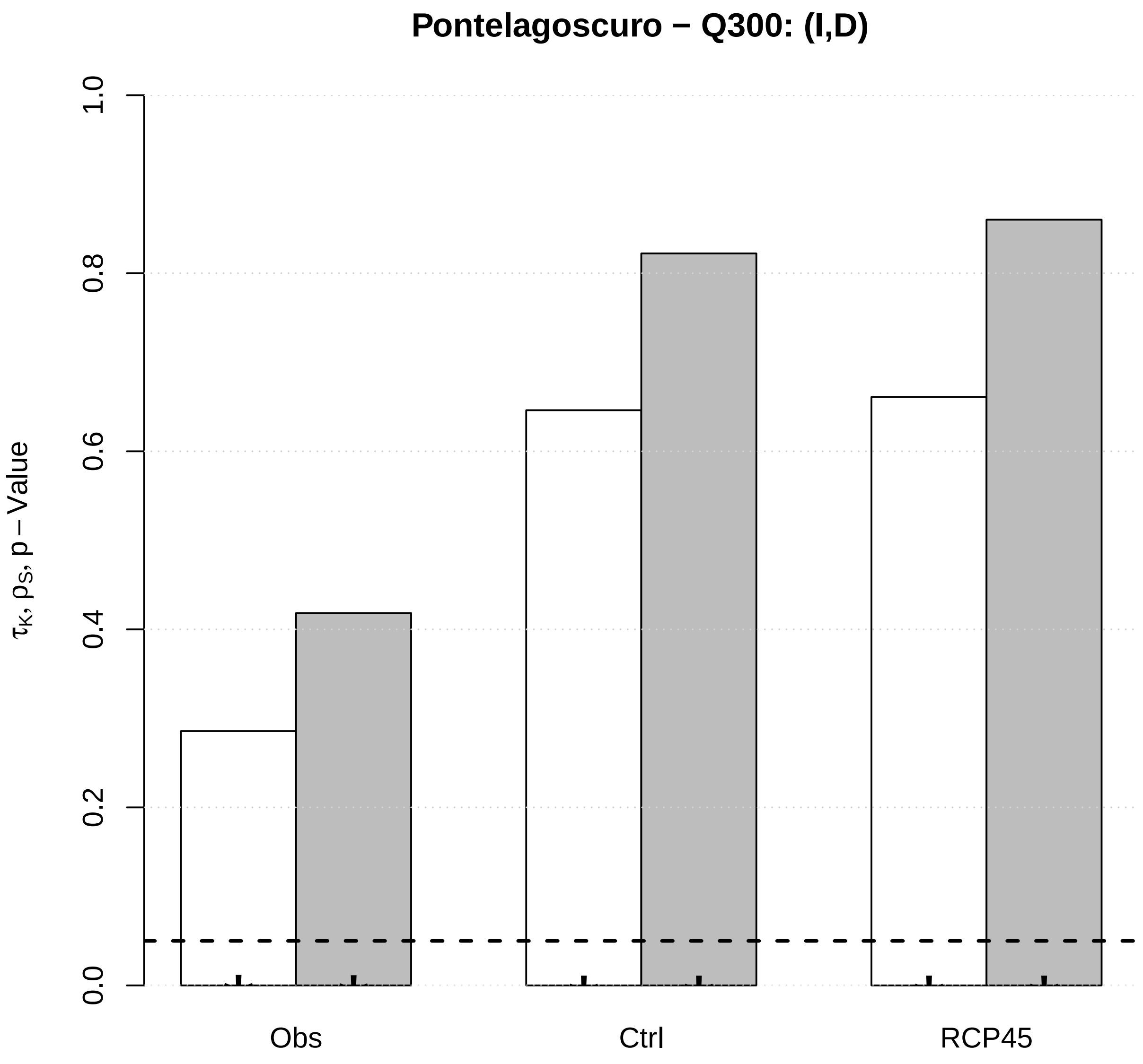

Figure 31: see text for explanation.

Drought analysis (bivariate): variables (I, D)

Data, Pseudo-observations, Empirical Copulas, Change-Point tests, Copula-Equality tests

In this Section, a bivariate statistical analysis of the drought occurrences will be presented. In particular, all the five available river sections (viz., Boretto, Borgoforte, Cremona, Piacenza, and Pontelagoscuro) will be considered: details can be found in the paper. Each sub-section contains the following plots, related to a single station, as indicated in the corresponding sub-section title.

Data, Pseudo-observations and Empirical Copulas. Considering the datasets of Observations (*Obs*), Control (*Ctrl*), and RCP4.5 (*RCP45*), the following plots are presented: the *top-left* panel concerns the Data, the *top-right* panel concerns the Pseudo-observations data, and the *bottom* panel concerns the isolines of the Empirical Copulas (averaged over all the N_R randomizations, with levels $0.1 : 0.1 : 0.9$).

Plotted are the data concerning the threshold Q_{300}: also reported are the sample sizes.

Change point and Equality tests. (*Top* panel) Shown are the box-plots of the p-Values of the Change-Point tests, over all the N_R randomizations, corresponding to the following data sets: Observations (*Obs*), Control (*Ctrl*), and RCP4.5 (*RCP45*). The distributions considered are: the marginal of the Intensity I (*F:I*), the marginal of the Duration D (*F:D*), the joint distribution of (I, D) (*F:(I,D)*), and the copula of (I, D) (*C:(I,D)*). The *dashed* horizontal line corresponds to the 5% reference level. Also reported are the sample sizes.

(*Bottom* panel) Shown are the box-plots of the p-Values of the Copula-Equality tests, over all the N_R randomizations, corresponding to the following pairs: the Observations–Control (*Obs,Ctrl*), the Observations–RCP4.5 (*Obs,RCP45*), and the Control–RCP4.5 (*Ctrl,RCP45*). The *dashed* horizontal line corresponds to the 5% reference level.

Plotted are the data concerning the threshold Q_{300}.

Boretto: Data, Pseudo-observations and Empirical Copulas

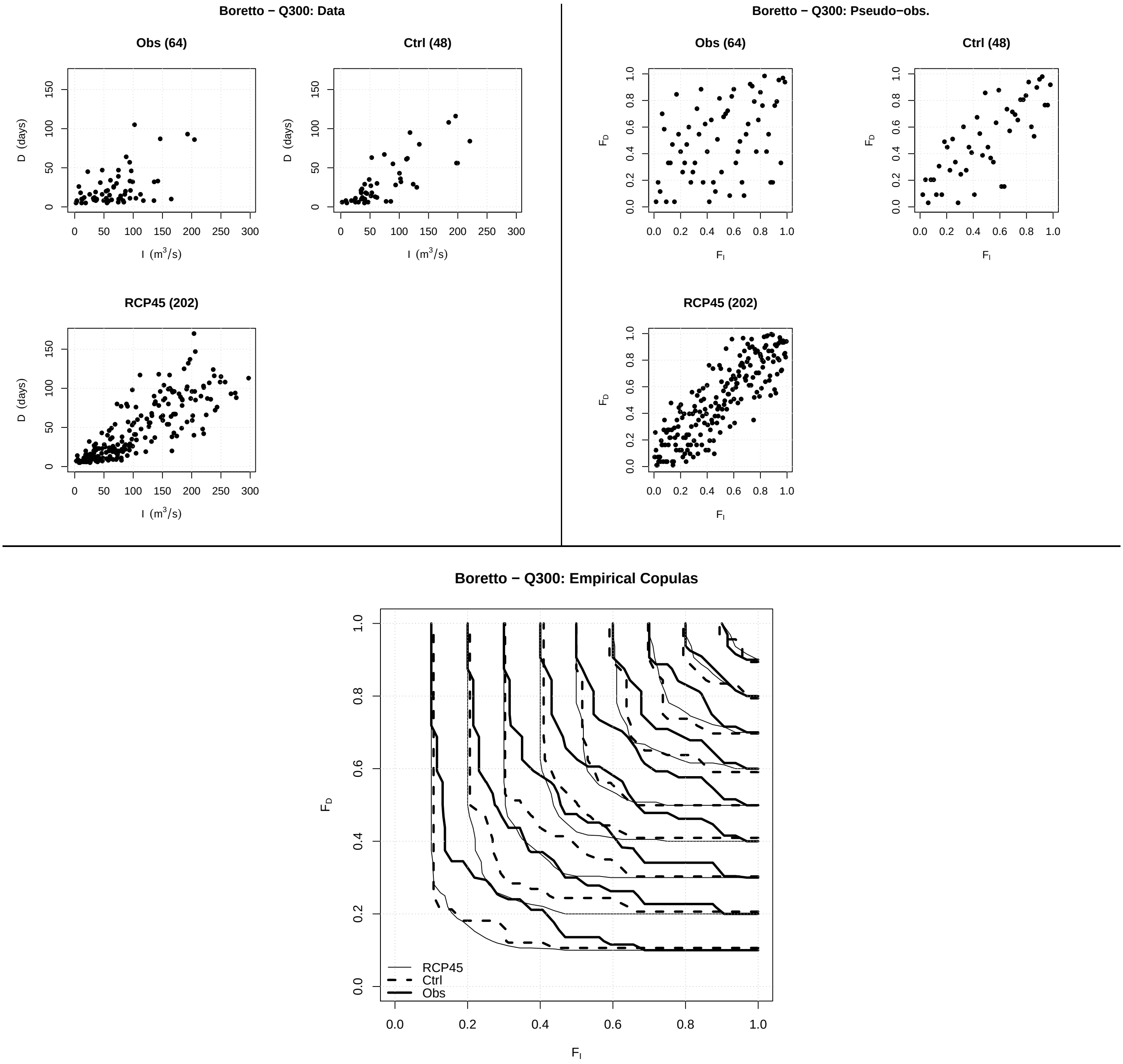

Figure 32: see text for explanation.

Boretto: Change-Point and Copula-Equality tests

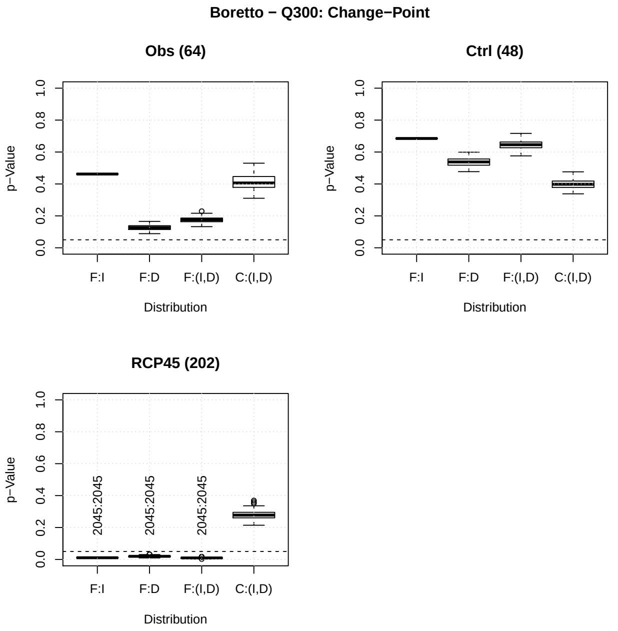

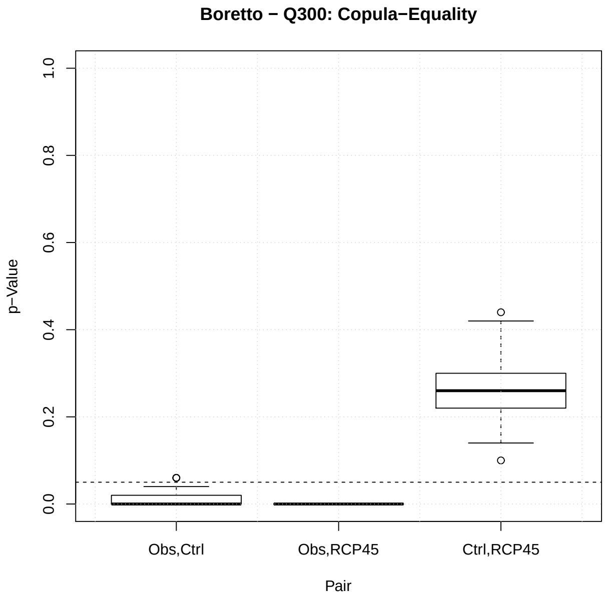

Figure 33: see text for explanation.

Borgoforte: Data, Pseudo-observations and Empirical Copulas

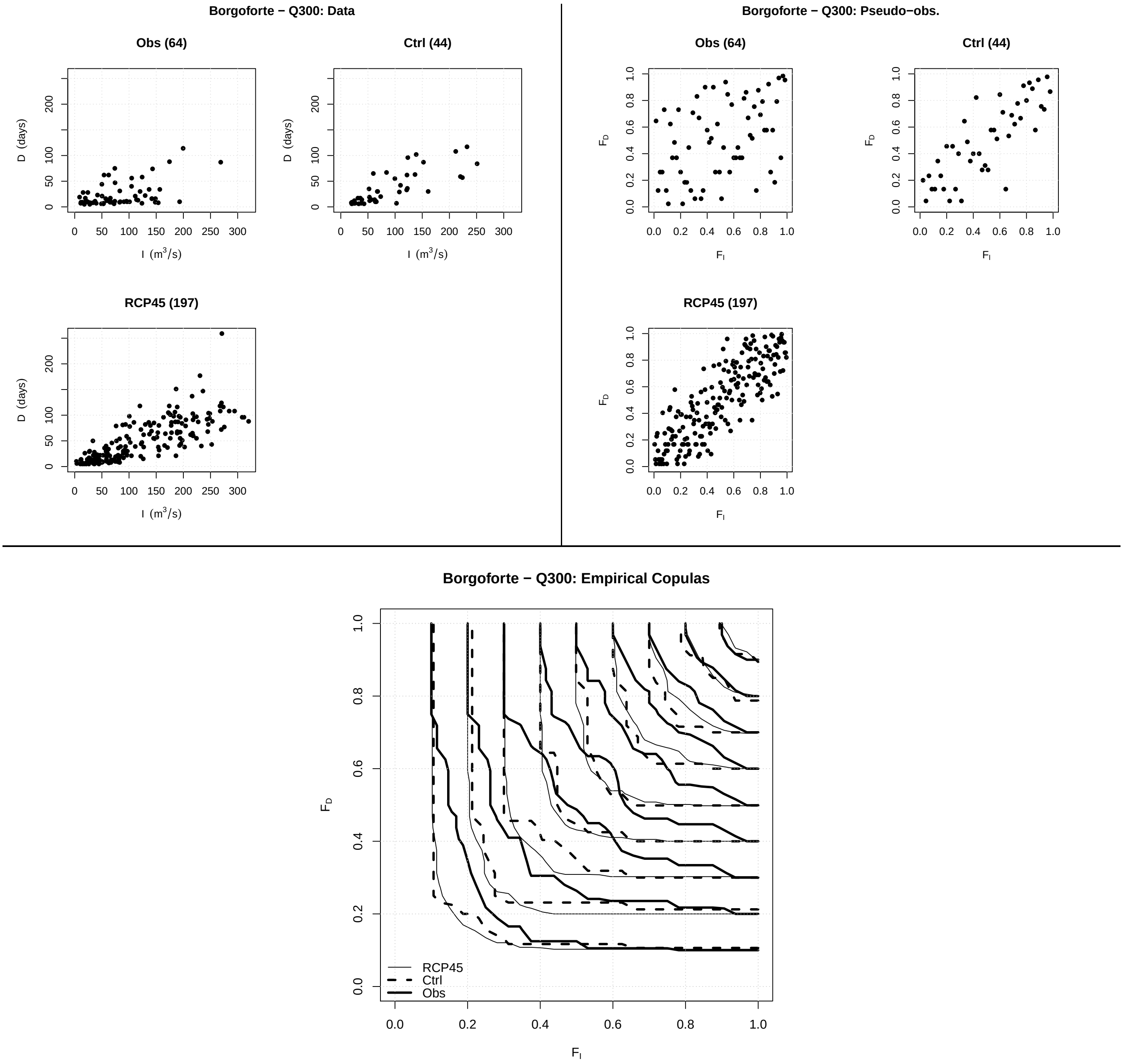

Figure 34: see text for explanation.

Borgoforte: Change-Point and Copula-Equality tests

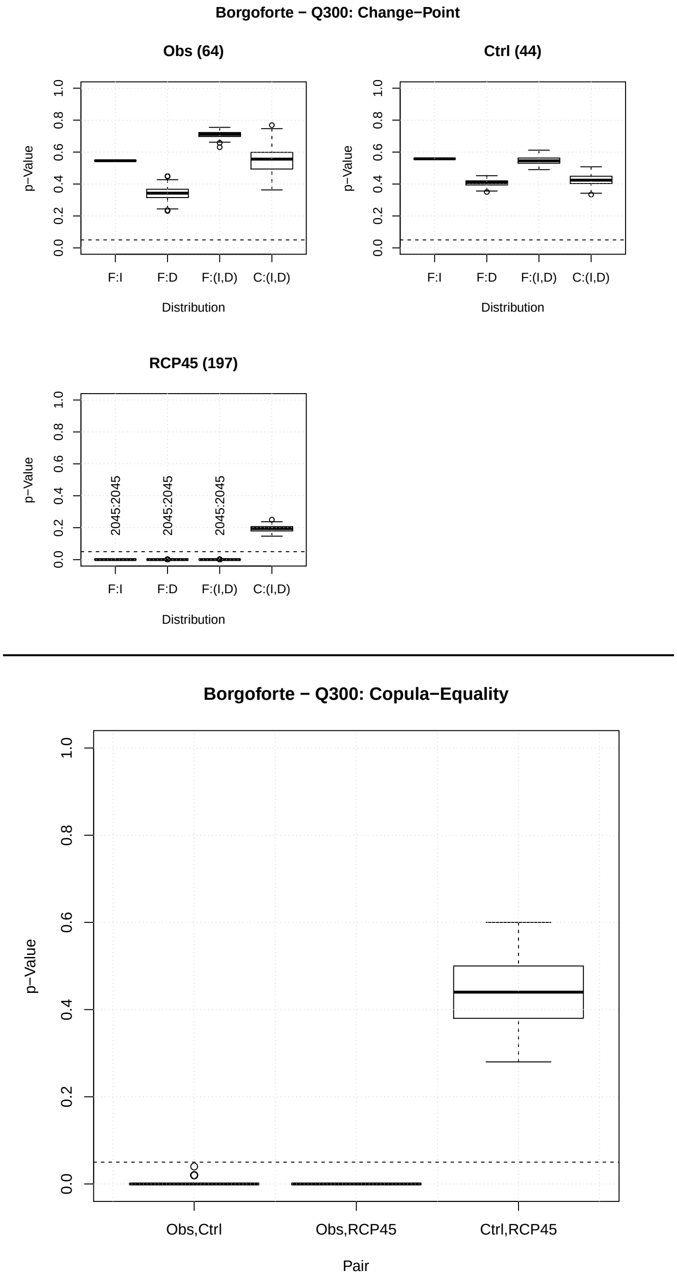

Figure 35: see text for explanation.

Cremona: Data, Pseudo-observations and Empirical Copulas

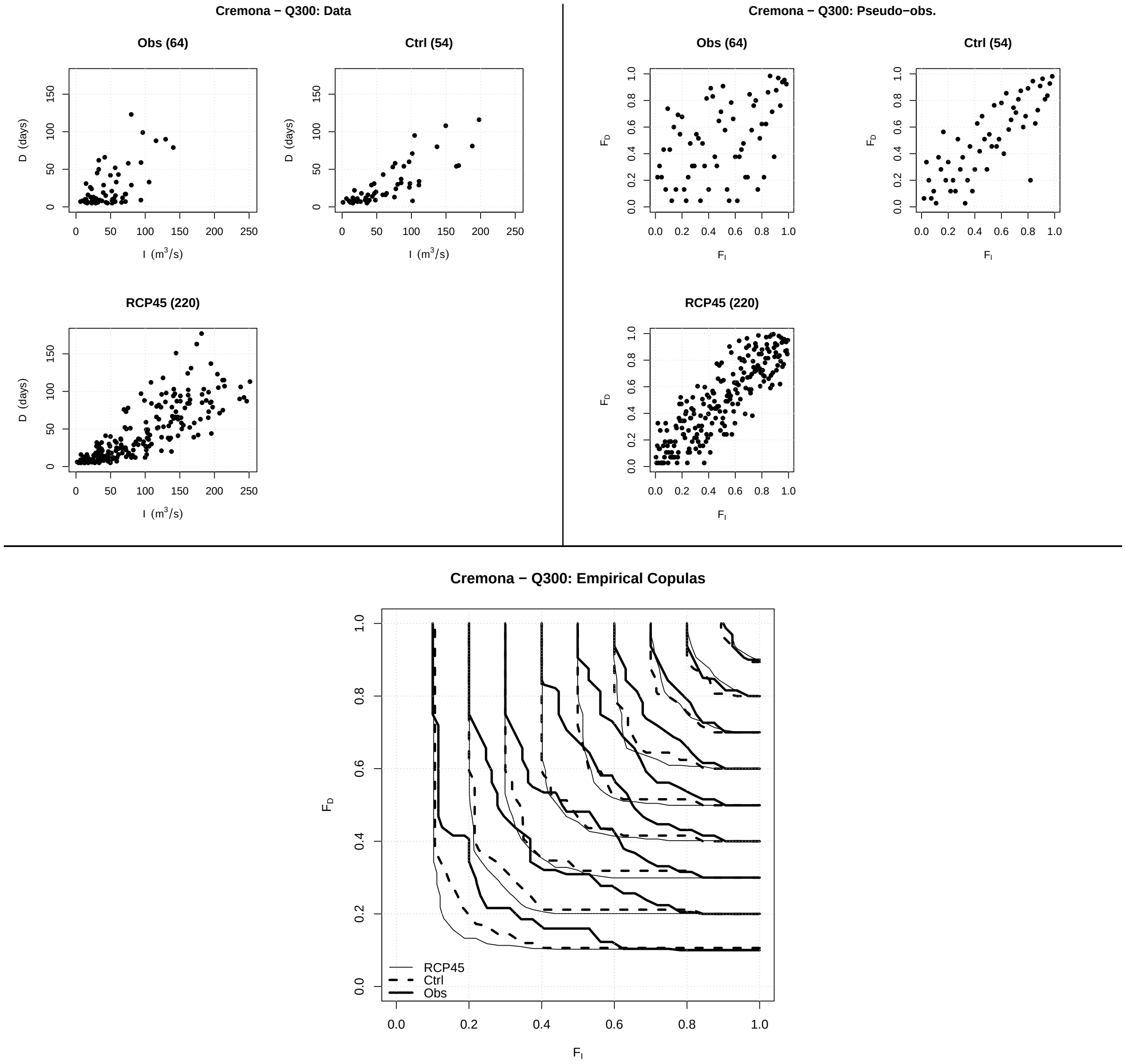

Figure 36: see text for explanation.

Cremona: Change-Point and Copula-Equality tests

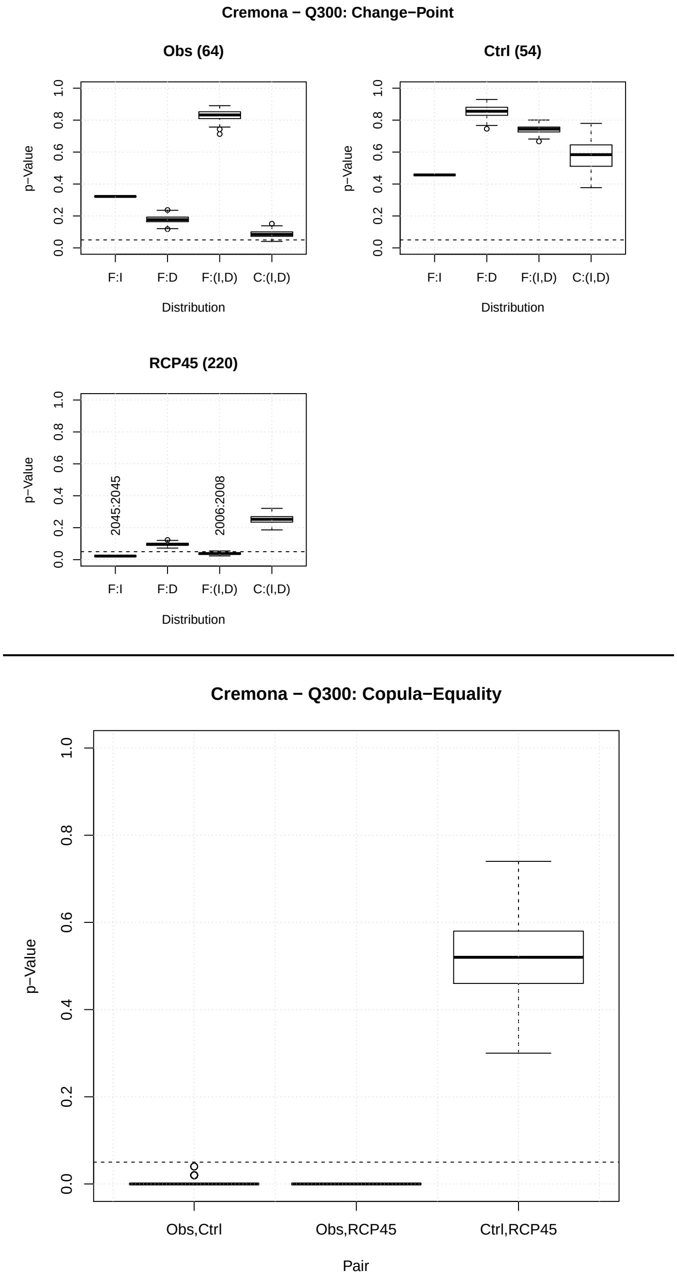

Figure 37: see text for explanation.

Piacenza: Data, Pseudo-observations and Empirical Copulas

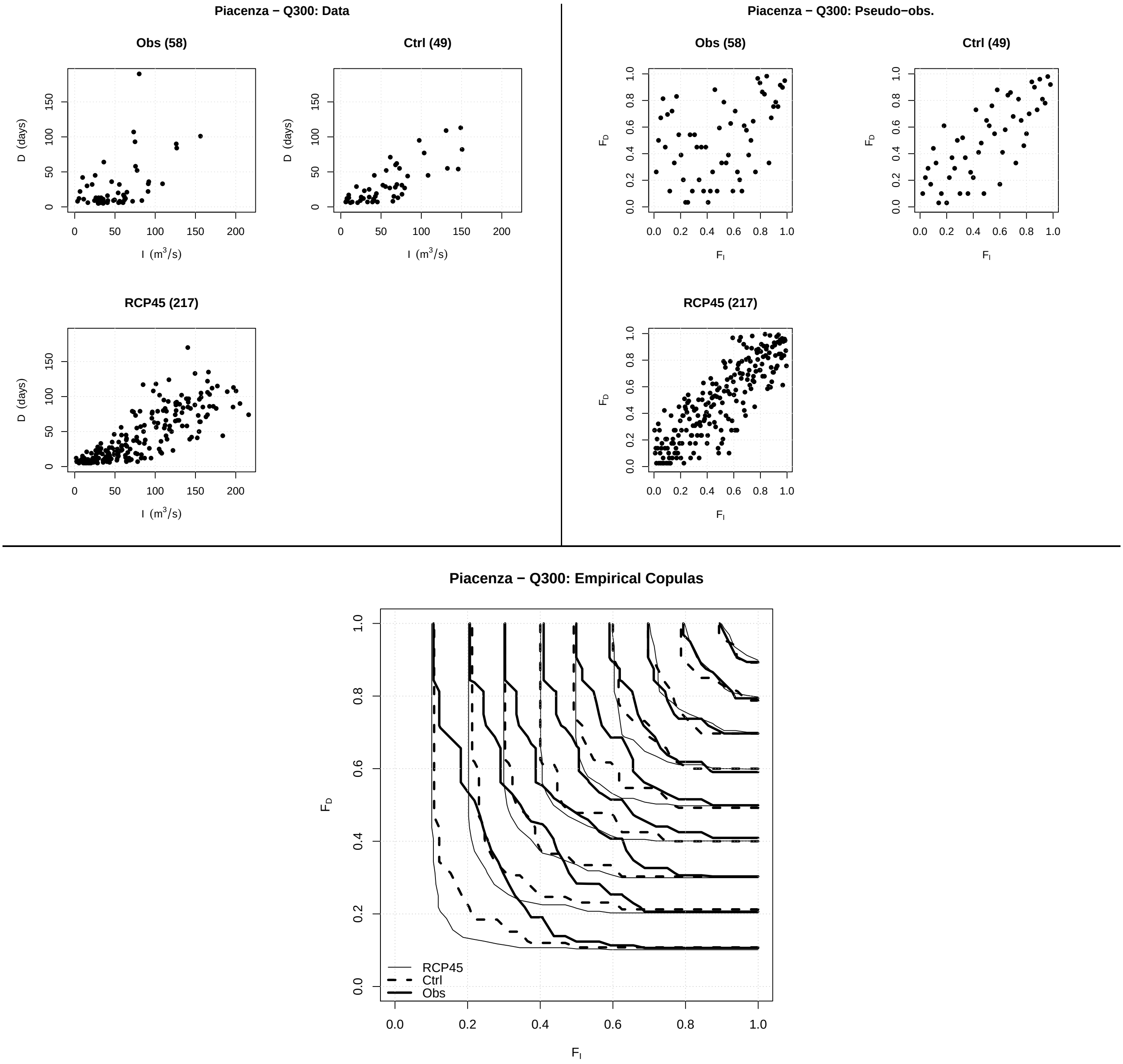

Figure 38: see text for explanation.

Piacenza: Change-Point and Copula-Equality tests

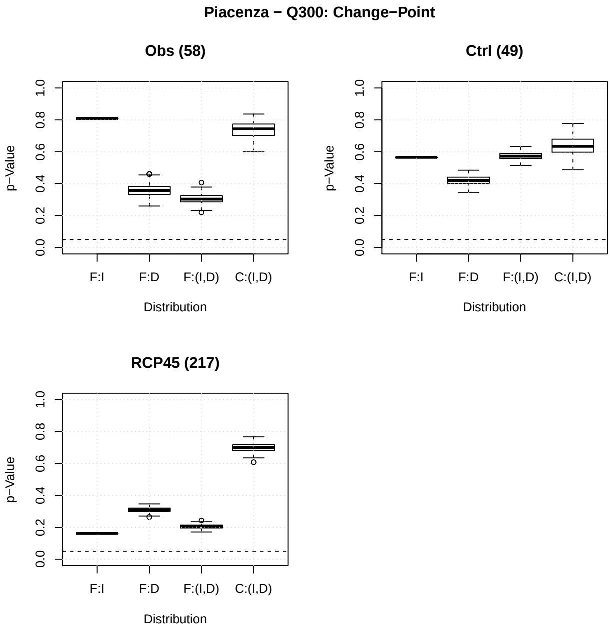

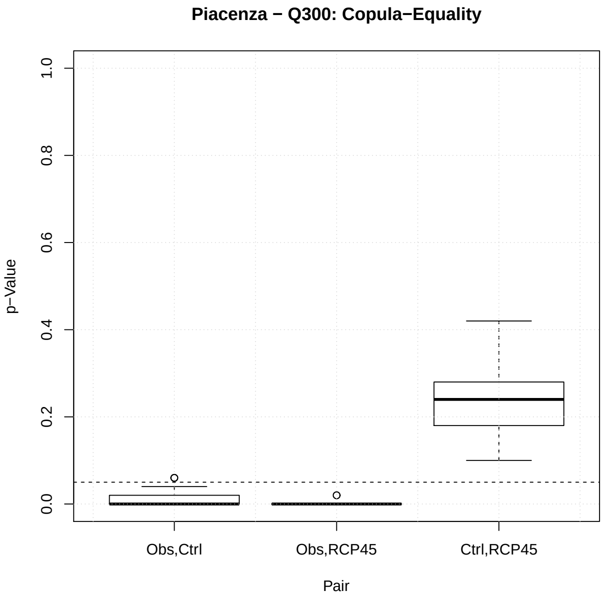

Figure 39: see text for explanation.

Pontelagoscuro: Data, Pseudo-observations and Empirical Copulas

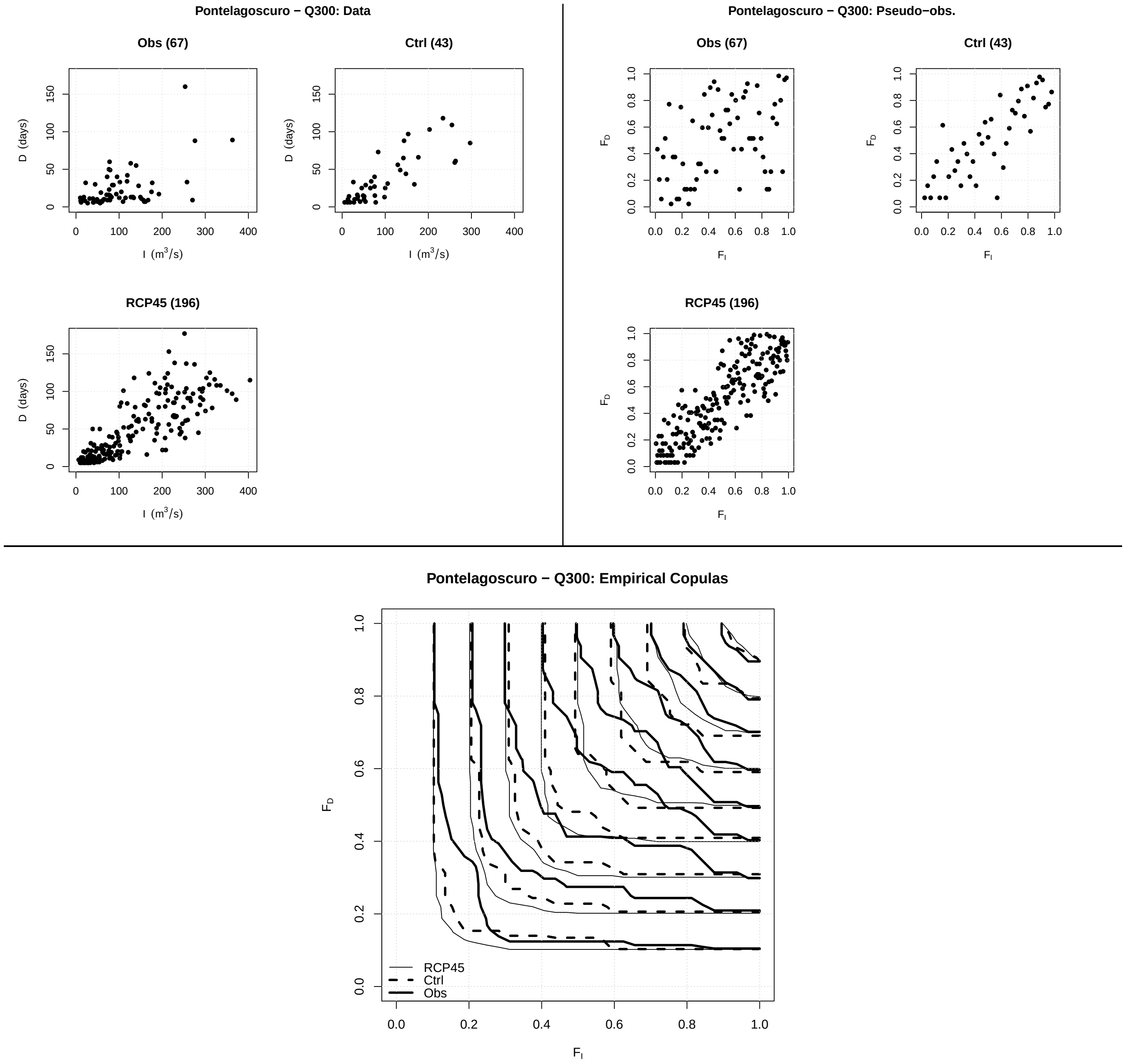

Figure 40: see text for explanation.

Pontelagoscuro: Change-Point and Copula-Equality tests

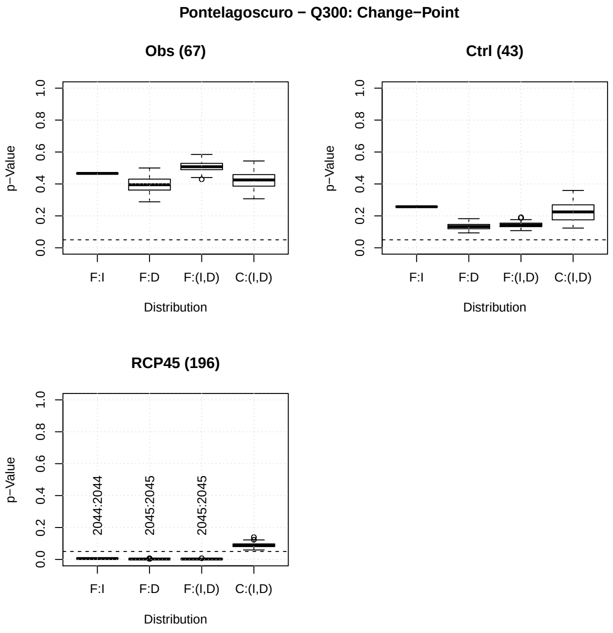

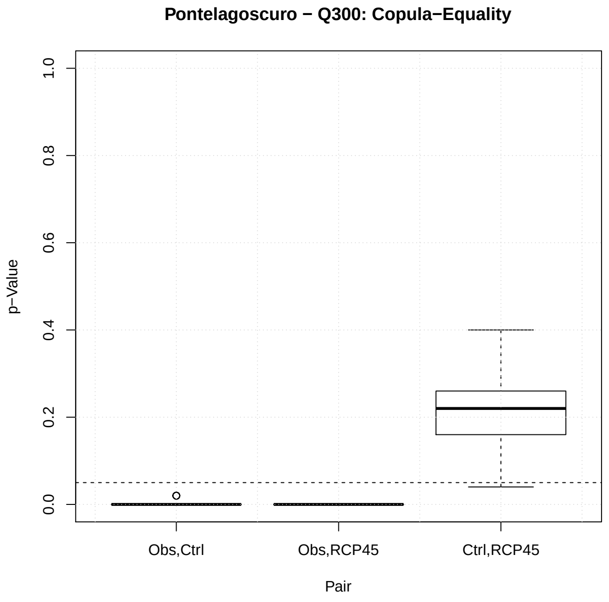

Figure 41: see text for explanation.
